# The Discovery of Phages in the Substantia Nigra and Its Implication for Parkinson’s Disease

**DOI:** 10.34133/research.0657

**Published:** 2025-04-30

**Authors:** Yun Zhao, Changxian Xiong, Bingwei Wang, Daotong Li, Jiarui Liu, Shizhang Wei, Yujia Hou, Yuan Zhou, Ruimao Zheng

**Affiliations:** ^1^ Department of Anatomy, Histology and Embryology, School of Basic Medical Sciences, Peking University, Beijing, China.; ^2^ Department of Biomedical Informatics, Center for Noncoding RNA Medicine, School of Basic Medical Sciences, Peking University, Beijing, China.; ^3^ Neuroscience Research Institute, Peking University, Beijing, China.; ^4^ Key Laboratory for Neuroscience of Ministry of Education, Peking University, Beijing, China.; ^5^ Key Laboratory for Neuroscience of National Health Commission, Peking University, Beijing, China.; ^6^ Beijing Life Science Academy, Beijing, China.

## Abstract

**Background:** A century ago, a mystery between a virus and Parkinson’s disease (PD) was described. Owing to the limitation of human brain biopsy and the challenge of electron microscopy in observing virions in human brain tissue, it has been difficult to study the viral etiology of PD. Recent discovery of virobiota reveals that viruses coexist with humans as symbionts. Newly developed transcriptomic sequencing and novel bioinformatic approaches for mining the encrypted virome in human transcriptome make it possible to study the relationship between symbiotic viruses and PD. Nevertheless, whether viruses exist in the human substantia nigra (SN) and whether symbiotic viruses underlie PD pathogenesis remain unknown. **Methods:** We collected current worldwide human SN transcriptomic datasets from the United States, the United Kingdom, the Netherlands, and Switzerland. We used bioinformatic approaches including viruSITE and the Viral-Track to identify the existence of viruses in the SN of patients. The comprehensive RNA sequencing-based virome analysis pipeline was used to characterize the virobiota in the SN. The Pearson’s correlation analysis was used to examine the association between the viral RNA fragment counts (VRFCs) and PD-related human gene sequencing reads in the SN. The differentially expressed genes (DEGs) in the SN between PD patients and non-PD individuals were used to examine the molecular signatures of PD and also evaluate the impact of symbiotic viruses on the SN. **Findings:** We observed the existence of viruses in the human SN. A dysbiosis of virobiota was found in the SN of PD patients. A marked correlation between VRFC and PD-related human gene expression was detected in the SN of PD patients. These PD-related human genes correlated to VRFC were named as the virus-correlated PD-related genes (VPGs). We identified 3 bacteriophages (phages), including the *Proteus* phage VB_PmiS-Isfahan, the *Escherichia* phage phiX174, and the *Lactobacillus* phage Sha1, that might impair the gene expression of neural cells in the SN of PD patients. The *Proteus* phage VB_PmiS-Isfahan was a common virus in the SN of patients from the United Kingdom, the Netherlands, and Switzerland. VPGs and DEGs together highlighted that the phages might dampen dopamine biosynthesis and weaken the cGAS-STING function. **Interpretation:** This is the first study to discover the involvement of phages in PD pathogenesis. A lifelong low symbiotic viral load in the SN may be a contributor to PD pathogenesis. Our findings unlocked the black box between brain virobiota and PD, providing a novel insight into PD etiology from the perspective of phage–human symbiosis.

## Introduction

Parkinson’s disease (PD) is characterized by loss of dopaminergic neurons in the substantia nigra (SN), involuntary shaking, and muscle rigidity [1–3]. Pathological hallmarks of PD include neuroinflammation [4–7], imbalanced protein homeostasis [8–11], oxidative stress [12–15], cell aging [16], and regulation of neurotransmitter and neuronal apoptosis [17–21]. Since the 1918 influenza pandemic, association between viruses and PD has been debated [22–28]. For a century, clinical evidence reveals that virus may cause PD-like symptoms [29,30]. Incidence of PD was lower in patients who received antiviral therapy [31,32]. Thus, there is a need to study the mechanism by which the virus may affect PD pathogenesis.

In recent years, the discovery of virobiota reveals that viruses coexist with humans as symbionts [33–42]. The human body is colonized with substantial communities of viruses [43], termed “virobiota” [44]. Bacteriophages (phages) are the most abundant viral entities in humans and also the major component of intestinal virobiota [45–47]. Phages can be disseminated throughout human tissues [48] and can cross the blood–brain barrier (BBB) to access the brain [49–54]. In mammalian cells, phages can enter organelles, maintain long-term residence, and affect cell function [55–58]. Phage genomes can be integrated into human chromosomes [59], and phage gene transcripts can be detected in human cells [60,61]. In the brain, phages can enter neural cells to cause neuroinflammation or neuronal death [55,56,62,63]. Together, these studies suggest that viruses/phages may affect the function of mammalian cells. However, whether viruses/phages exist in the human SN and whether viruses/phages may be linked to PD pathogenesis remain unknown.

Next-generation sequencing becomes novel approach to identify and characterize virobiota in tissues [64]. Sequencing of viral mRNA fragments that encrypted in human transcriptome reveal viral involvement in human diseases [65]. Newly developed bioinformatic approaches, viruSITE [66] and Viral-Track [64], are designed for mining the encrypted virome in transcriptome of host tissues. Correlation analysis between viral RNA fragment counts (VRFCs) and human host gene sequencing reads can uncover the relationship between virus and human diseases [67,68]. Genome-wide microarray datasets of human SN also contribute to characterize PD-related gene expression [69–74]. Therefore, worldwide RNA-sequencing (RNA-seq)/microarray studies performed on autopsy SN samples of PD patients can provide resources for exploring the link between the SN virobiota and PD pathogenesis. In this study, by using transcriptomic datasets of SN samples from Geneva University Hospitals, Netherlands Brain Bank, and Parkinson’s UK Brain Bank, we identified a viral existence in the SN and a strong correlation between VRFCs of phages and PD-related human gene expression in the SN of PD patients. Our findings discovered that brain virobiota may underlie PD pathogenesis.

## Results

### Characteristics of RNA-seq/microarray datasets of the SN of PD patients

**Fig. 1.**
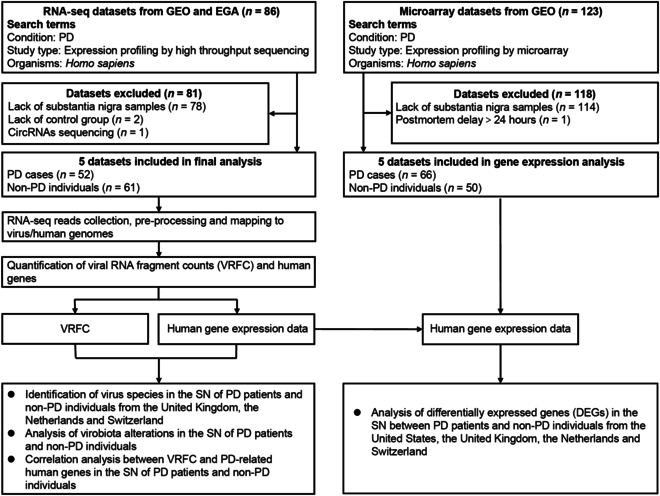
Enrollment and analysis of datasets.

**Fig. 2.**
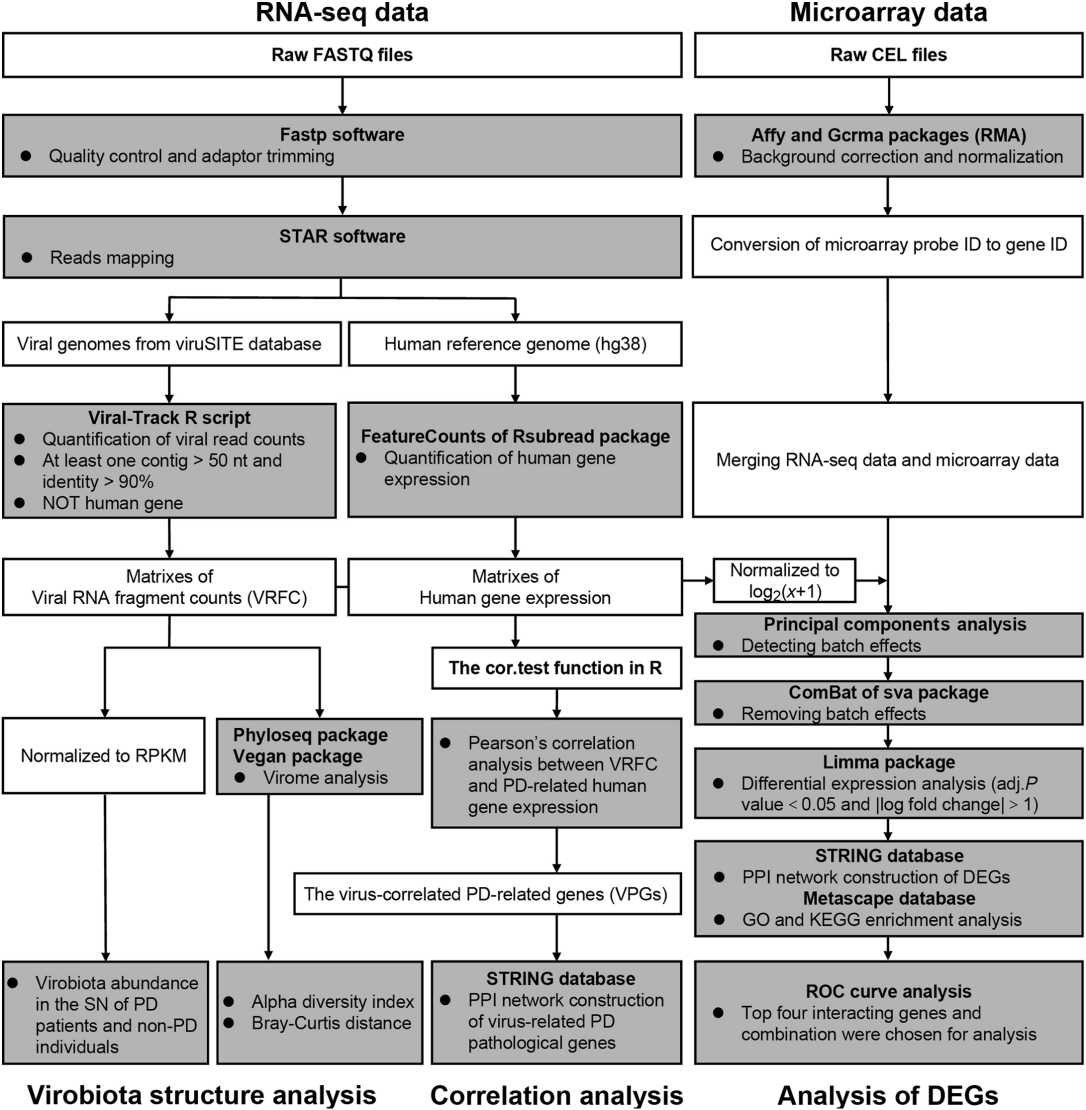
Flowchart of bioinformatic analysis pipelines.

Worldwide PD brain RNA-seq datasets from the United Kingdom, the Netherlands, and Switzerland were collected (Fig. 1); microarray datasets from the United States, the United Kingdom, and the Netherlands were enrolled (Tables S1 and S2). There are comparable PD prevalence, average life expectancy, and gender composition among these countries. To ensure comparability and homogeneity of raw data, “Combat” method was used to correct batch effects, when required (Fig. 2).

### Viral sequences were detected in the human SN

**Fig. 3.**
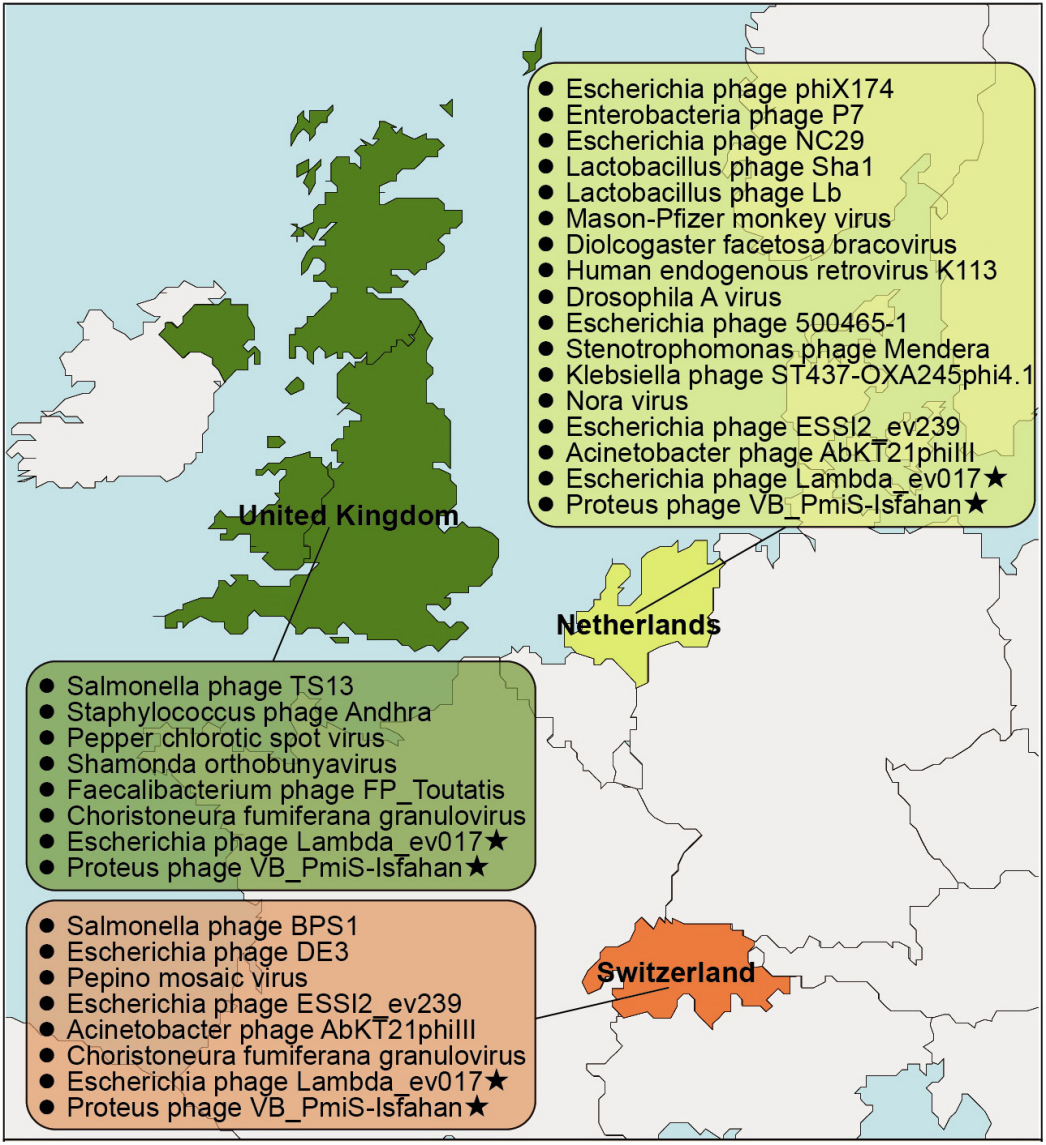
Identification of viral species in the SN samples from the brain banks of the United Kingdom, the Netherlands, and Switzerland.

Viral mRNA fragments were detected in the SN samples. These viruses included the phages that host gut microbiota, the viruses that host primates, and the viruses that host plants and arthropods (Table and Table S3). The *Proteus* phage VB_PmiS-Isfahan and the *Escherichia* phage Lambda_ev017 were common viruses in the SN of patients from these countries, showing that the viral population distribution may be geographically related (Fig. 3). These findings revealed an existence of viruses in the human SN, suggesting that the human SN may be colonized by commensal virobiota.

**Table.** Viruses of nearest match identified in the SN of PD patients

Viral family	Virus of nearest match	% Nucleotide identity	Contig size range (nucleotides)	Main reported host	Baltimore classification
Siphoviridae	*Escherichia* phage Lambda_ev017	100	35–79	*Escherichia*	I: dsDNA
Siphoviridae	*Proteus* phage VB_PmiS-Isfahan	100	50–510	*Proteus mirabilis*	I: dsDNA
Siphoviridae	*Lactobacillus* phage Sha1	100	53–122	*Lactobacillus* sp.	I: dsDNA
Siphoviridae	*Escherichia* phage DE3	100	52–150	*Escherichia coli* BL21(DE3)	I: dsDNA
Casjensviridae	*Salmonella* phage TS13	100	43–68	*Salmonella enterica*	I: dsDNA
Casjensviridae	Salmonella phage BPS1	97	33–52	*Salmonella enterica* subsp	I: dsDNA
Myoviridae	*Faecalibacterium* phage FP_Toutatis	100	47–117	*Faecalibacterium prausnitzii*	I: dsDNA
Peduoviridae	*Escherichia* phage ESSI2_ev239	100	35–93	*Escherichia*	I: dsDNA
Peduoviridae	*Escherichia* phage 500465-1	100	87–364	*Escherichia coli*	I: dsDNA
Peduoviridae	*Klebsiella* phage ST437-OXA245phi4.1	100	45–134	*Klebsiella pneumoniae*	I: dsDNA
Retroviridae	Human endogenous retrovirus K113	100	53–136	*Homo sapiens*	VI: ssRNA-RT
Retroviridae	Mason–Pfizer monkey virus	100	61–68	*Macaca mulatta*	VI: ssRNA-RT
Microviridae	*Escherichia* phage phiX174	100	53–5,386	*Escherichia coli*	II: ssDNA (+)
Microviridae	*Escherichia* phage NC29	99	92	*Escherichia*	II: ssDNA (+)
Autographiviridae	*Acinetobacter* phage AbKT21phiIII	94	52–63	*Acinetobacter baumannii*	I: dsDNA
Rountreeviridae	*Staphylococcus* phage Andhra	95	29–56	*Acanthamoeba castellanii*	I: dsDNA
Polydnaviriformidae	*Diolcogaster facetosa* bracovirus	100	55–93	*Glyptapanteles flavicoxis*	I: dsDNA
Baculoviridae	*Choristoneura fumiferana* granulovirus	100	36–77	*Choristoneura fumiferana*	I: dsDNA
Tospoviridae	Pepper chlorotic spot virus	93	71	Peppers	V: ssRNA (−)
Peribunyaviridae	Shamonda orthobunyavirus	94	87	*Bos taurus*	V: ssRNA (−)
Alphaflexiviridae	Pepino mosaic virus	96	67	*Solanum lycopersicum*	IV: ssRNA (+)
NA	Enterobacteria phage P7	100	35–360	*Escherichia coli*	I: dsDNA
NA	*Stenotrophomonas* phage Mendera	96	56	*Stenotrophomonas maltophilia*	I: dsDNA
NA	*Lactobacillus* phage Lb	100	73–116	*Lactobacillus brevis* ATCC 367	I: dsDNA
NA	Nora virus	98	84–113	*Drosophila melanogaster*	IV: ssRNA (+)
NA	*Drosophila* A virus	99	77–176	*Drosophila melanogaster*	IV: ssRNA (+)

### Dysbiosis of virobiota in the SN of PD patients

**Fig. 4.**
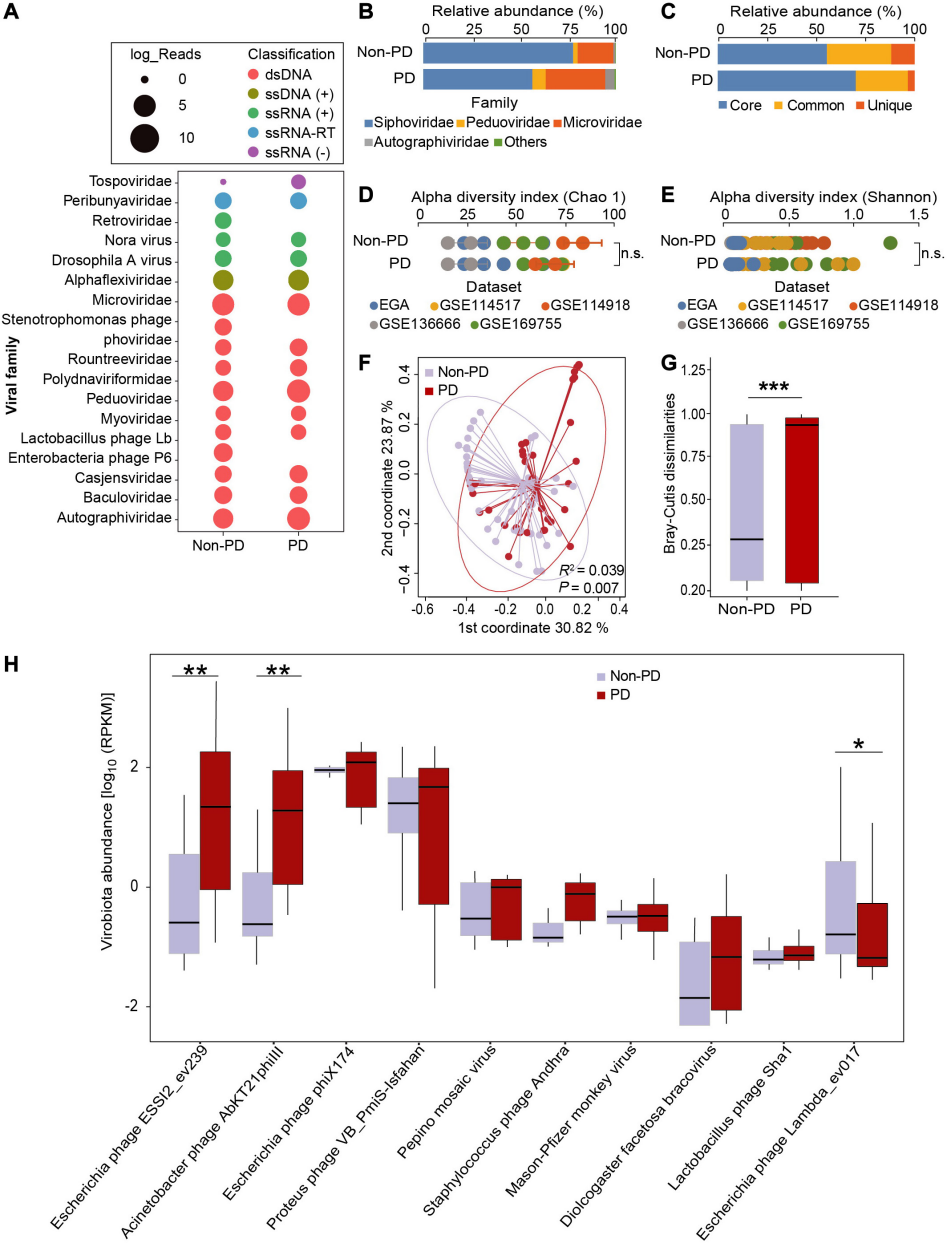
Dysbiosis of virobiota in the SN of PD patients. (A) Bubble plot showing the read abundances of different viral families in the SN virome. Read counts are log_2_-transformed and represented by the point sizes. (B) Proportion of viral families in the SN virome. (C) Proportion of core, common, and unique viral species in the SN virome. The core species, common species, and unique species correspond to viral species shared among >80%, 30% to 80%, and <30% of studied group, respectively. (D and E) Comparison of α-diversity of the SN virome based on Chao1 richness index (D) and Shannon diversity (E). n.s., not significant. Statistical significance was determined by *t* test. (F) Principal coordinate analysis (PCoA) plot of the Bray–Curtis distance showing the PD patients and non-PD individuals. Statistical significance for the Bray–Curtis distance was determined by PERMANOVA with permutations done 999 times. (G) Comparison of within-group SN virome Bray–Curtis dissimilarities between PD patients and non-PD individuals. Statistical significance was determined by *t* test. ***P < 0.001. (H) Differential viral taxa between PD and non-PD at the species level. Differentially enriched viral species were determined by DESeq2 analysis. For viral abundance box plots, the boxes extend from the first to the third quartile (25th to 75th percentiles), with the median depicted by a horizontal line. RPKM, reads per kilobase per million mapped reads. *P < 0.05 and **P < 0.01.

In SN samples, 11 viral families were detected (Fig. 4A). In PD patients, viral families Peduoviridae, Microviridae, and Autographiviridae were enriched, whereas viral family Siphoviridae was diminished, as compared with non-PD individuals (Fig. 4B). To explore difference in composition of the SN virobiota between groups, we quantified the presence ratio of core, common, and unique viral species (corresponding to viral species shared among >80%, 30% to 80%, and <30% of the individuals, respectively). In the SN of PD patients, the core species accounted for higher proportion, whereas unique species was diminished (Fig. 4C). Richness (Chao1) and diversity (Shannon) of virome in the SN did not differ between groups (Fig. 4D and E). Composition of virome in the SN of 2 groups was separated into 2 distinct clusters. Viral community dissimilarity among PD patients was higher than that of non-PD individuals (Fig. 4F and G). Analysis of differentially present taxa at the species level shows a remarkable difference in viral community structures between groups (Fig. 4H). Together, these findings uncovered a dysbiosis in the SN virome of PD patients, suggesting that altered species proportion and virobiota dysbiosis may be associated with PD pathogenesis.

### A strong negative correlation between VRFC of the *Proteus* phage VB_PmiS-Isfahan and human PD-related gene sequencing reads in the SN of PD patients

**Fig. 5.**
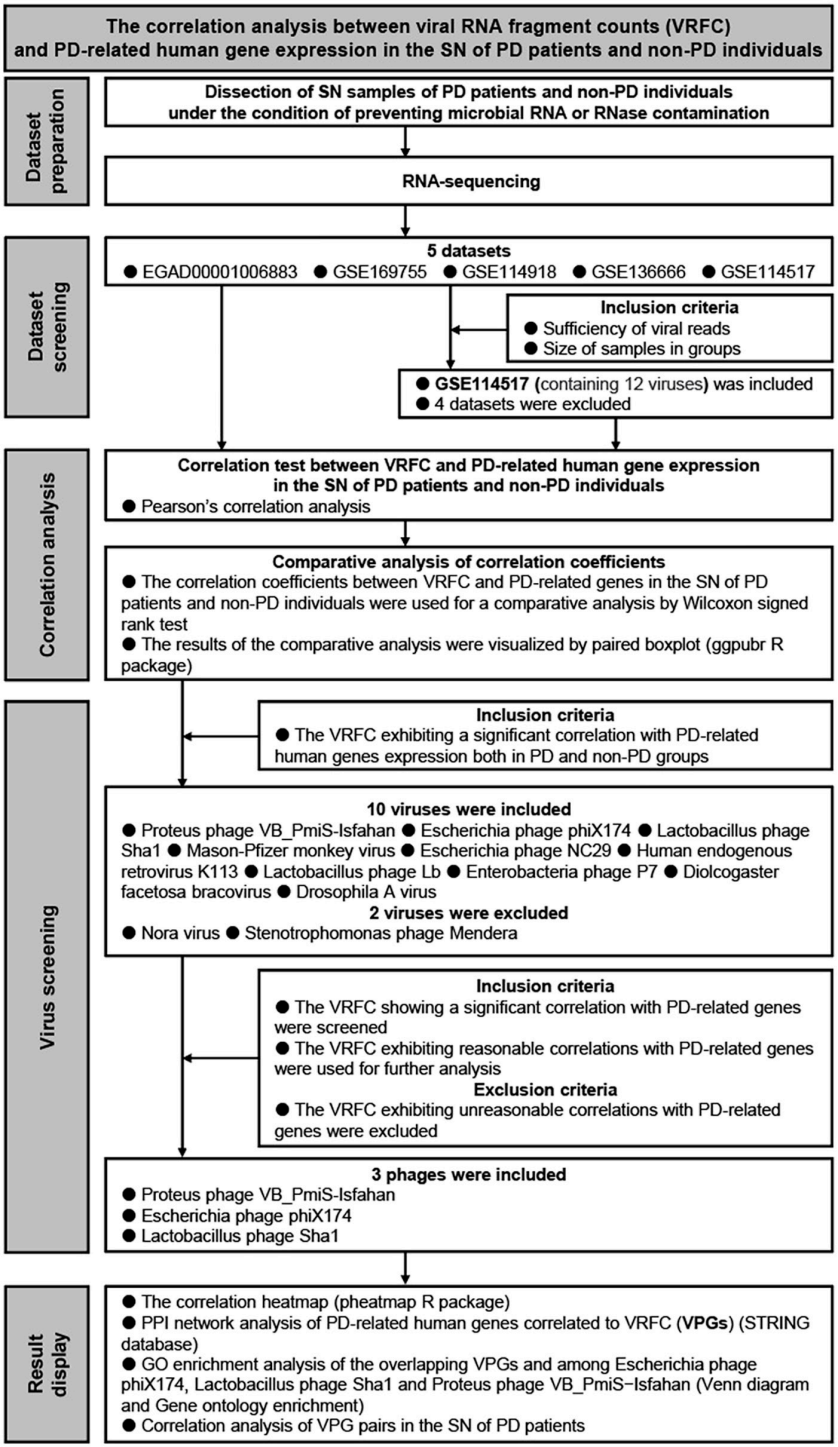
Enrollment of datasets and correlation analysis between VRFC and PD-related human gene expression.

**Fig. 6.**
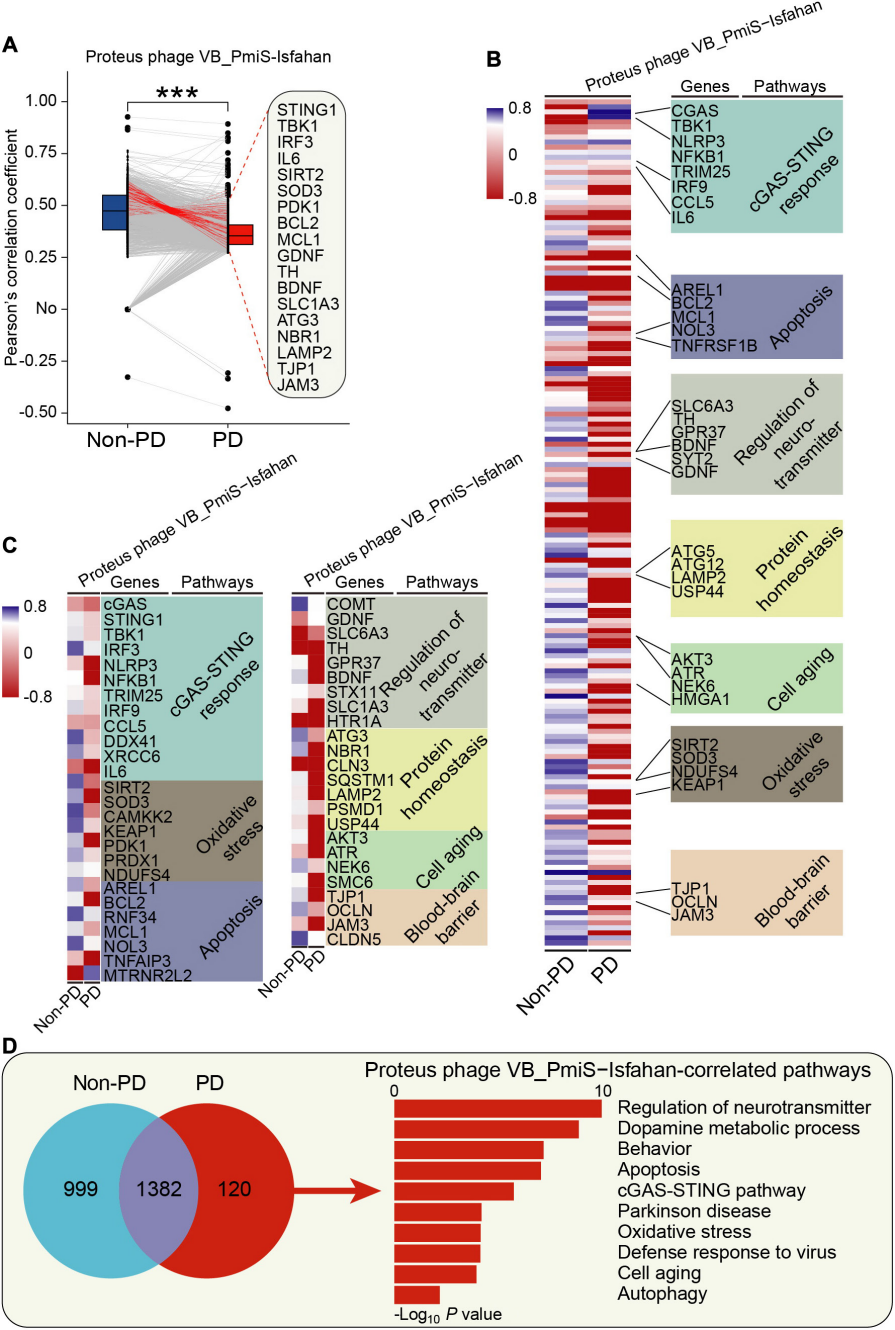
A strong negative correlation between VRFC of the *Proteus* phage VB_PmiS-Isfahan and PD-related human gene expression in the SN of PD patients. (A) Paired box plots showing changes of the correlations between VRFC of the *Proteus* phage VB_PmiS-Isfahan and PD-related human gene expression in the SN of PD patients and non-PD individuals. Top and bottom edges represent the first and third quartiles, respectively; the center line represents the median. The *P* values were calculated using the Wilcoxon matched-pairs test. ***P < 0.001. (B) Hierarchical clustered heatmap of correlation profiles between VRFC of the *Proteus* phage VB_PmiS-Isfahan and PD-related human gene expression in the SN of PD patients and non-PD individuals. In the heat map, each column represents a phage, and each row represents a human gene. Red denotes negative correlation, and blue denotes positive correlation. (C) Heatmap of correlation profiles between VRFC of the *Proteus* phage VB_PmiS-Isfahan and expression of PD-related genes in the SN of PD patients and non-PD individuals. (D) Venn diagram showing the overlapping PD-related genes and pathways between non-PD individuals and PD patients of the *Proteus* phage VB_PmiS-Isfahan.

To explore whether gene activity of symbiotic viruses in the SN may underlie PD pathogenesis, correlation analysis between VRFCs and PD-related human gene expression was performed (Fig. 5). Based on GSE114517 dataset, a strong negative correlation between VRFCs of phages and PD-related human gene expressions (tyrosine hydroxylase, TH, a dopamine synthetase, etc.) was detected in the SN of PD patients; pathways affected by these phages were similar in PD pathophysiology, although the phages belong to different genera and families (Fig. S2A to C and Tables S4 to S6). We named these human PD-related genes that are correlated to the VRFC as “the virus-correlated PD-related genes (VPGs)”. The VPGs suppressed by the phages were enriched for the PD-related pathways including cGAS-STING response, oxidative stress, and apoptosis (Fig. S2D and E and Table S7). The protein–protein interaction (PPI) network revealed 322 pairs of interactions among these VPGs (Fig. S1A). The top 19 VPGs with more than 15 interactions were shown in bar plot (Fig. S1B). A strong correlation coefficient among the VPGs was observed in PD patients (Fig. S1C). Venn analysis revealed a large overlap of 1,313 genes among the VPGs affected by 3 phages (Fig. S2F). The *Proteus* phage VB_PmiS-Isfahan was a common virus in the SN of patients from these countries. Likewise, based on all 5 datasets, similar results between VRFCs of the *Proteus* phage VB_PmiS-Isfahan and PD-related human gene expression in the SN of PD patients were also observed (Fig. 6). Table S8 outlines the key phages associated with PD pathogenesis and phage-affected PD pathways. No significant differences were observed in VRFC of these phages across groups, indicating that gene expressions of these viruses were comparable between datasets (Fig. S3). Together, these suggest that phages may disrupt human gene expression in the SN to inhibit dopamine biosynthesis, neural growth factors, and antiviral factors. Symbiotic virobiota may increase the risk of PD.

### DEGs reveal a potential association between symbiotic viruses and PD pathogenesis

**Fig. 7.**
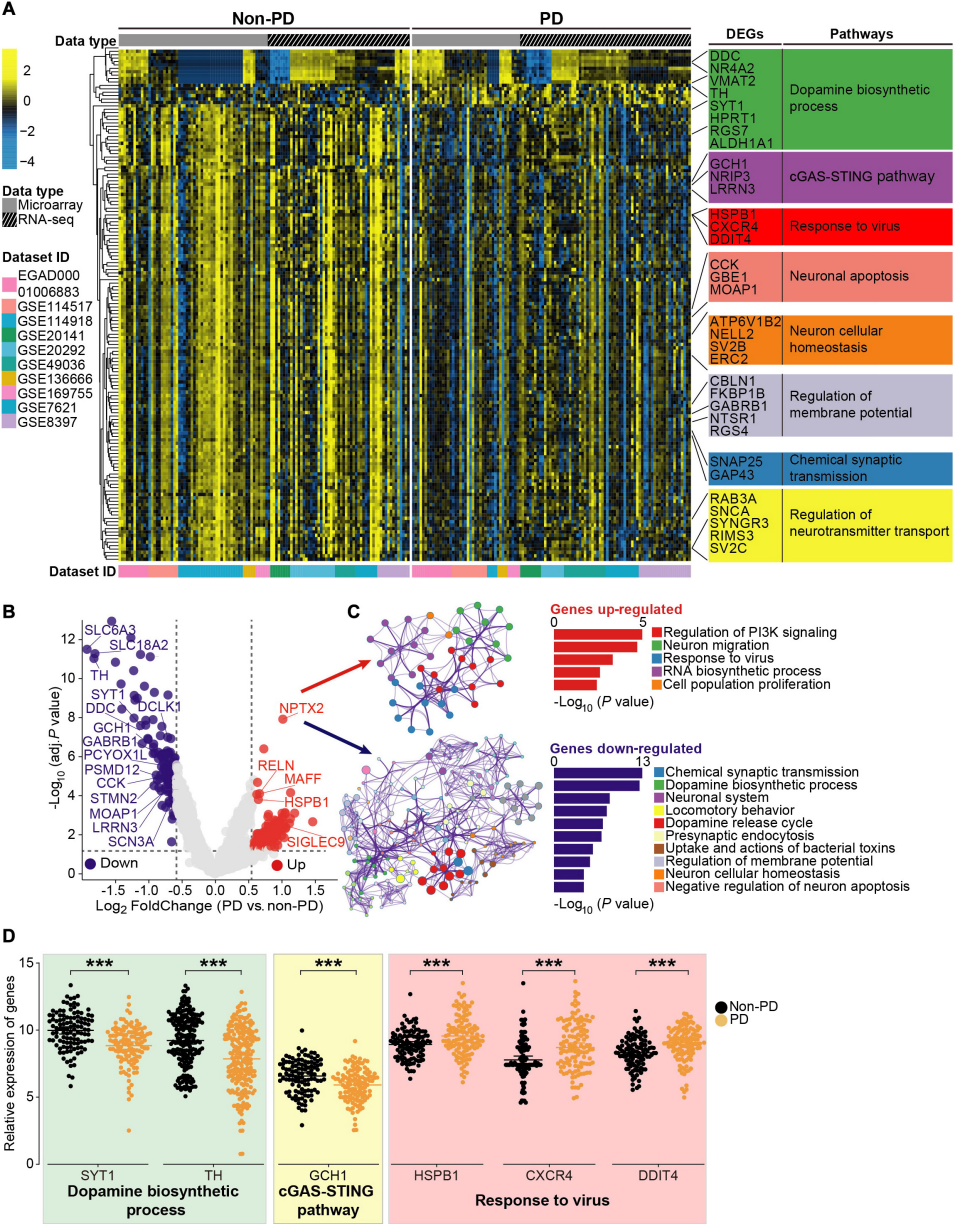
DEGs reveal a potential association between symbiotic phages and PD pathogenesis. (A) Hierarchical clustered heatmap of DEGs in the SN of PD patients and non-PD individuals. (B) Volcano plot displays DEGs in the SN of PD patients compared with non-PD individuals. Up-regulated genes are colored in red, down-regulated genes are colored in blue, and insignificantly altered genes are colored in gray. (C) GO and KEGG enrichment analysis of DEGs. (D) Expression level of genes. The total relative expression levels of genes are shown as median and 95% confidence interval. The relative expression level of genes in the SN of PD patients (*n* = 118) and non-PD individuals (*n* = 111). Statistical significance was determined by Wilcoxon test, ***P < 0. 001.

**Fig. 8.**
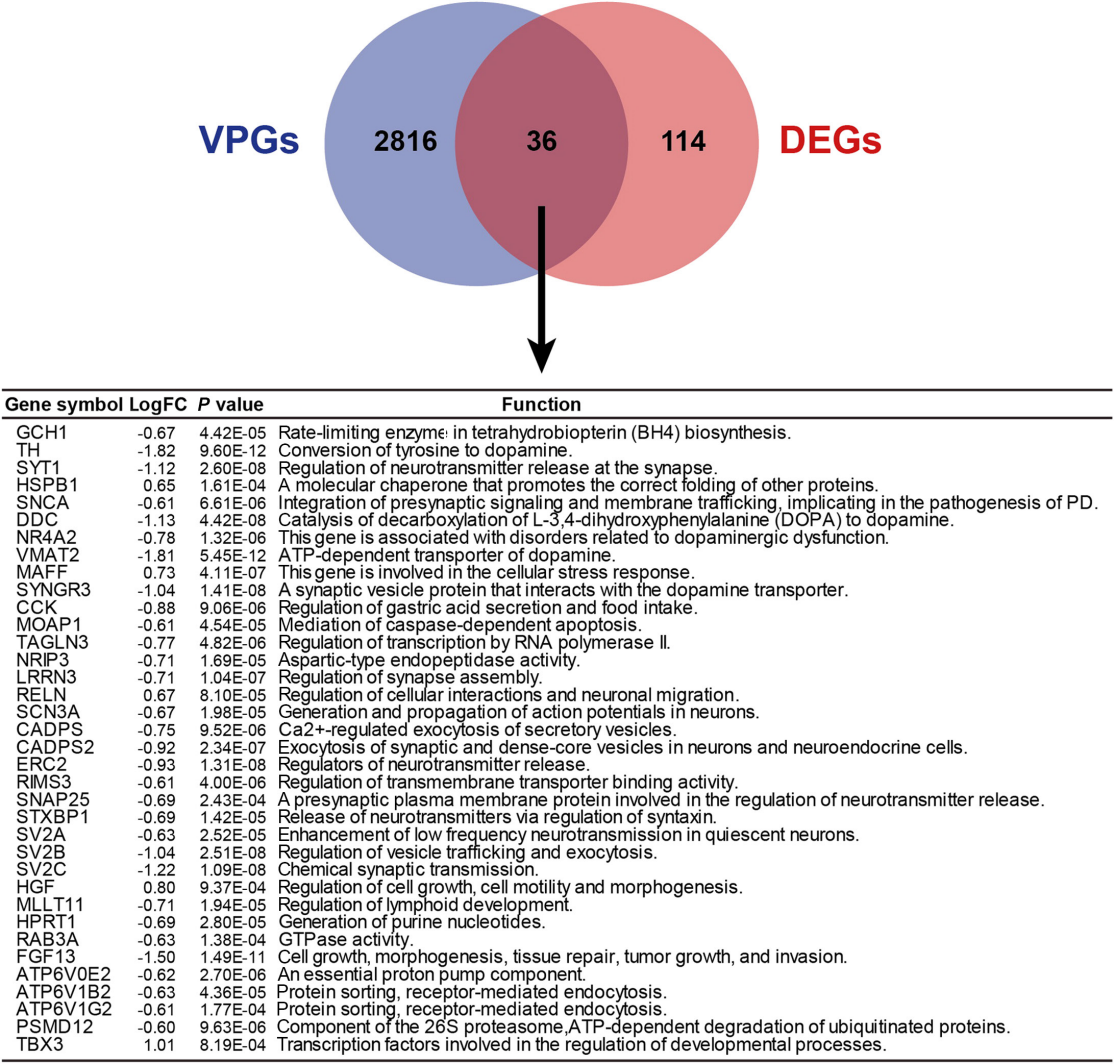
The overlapping genes between VPGs and DEGs in the SN.

**Fig. 9.**
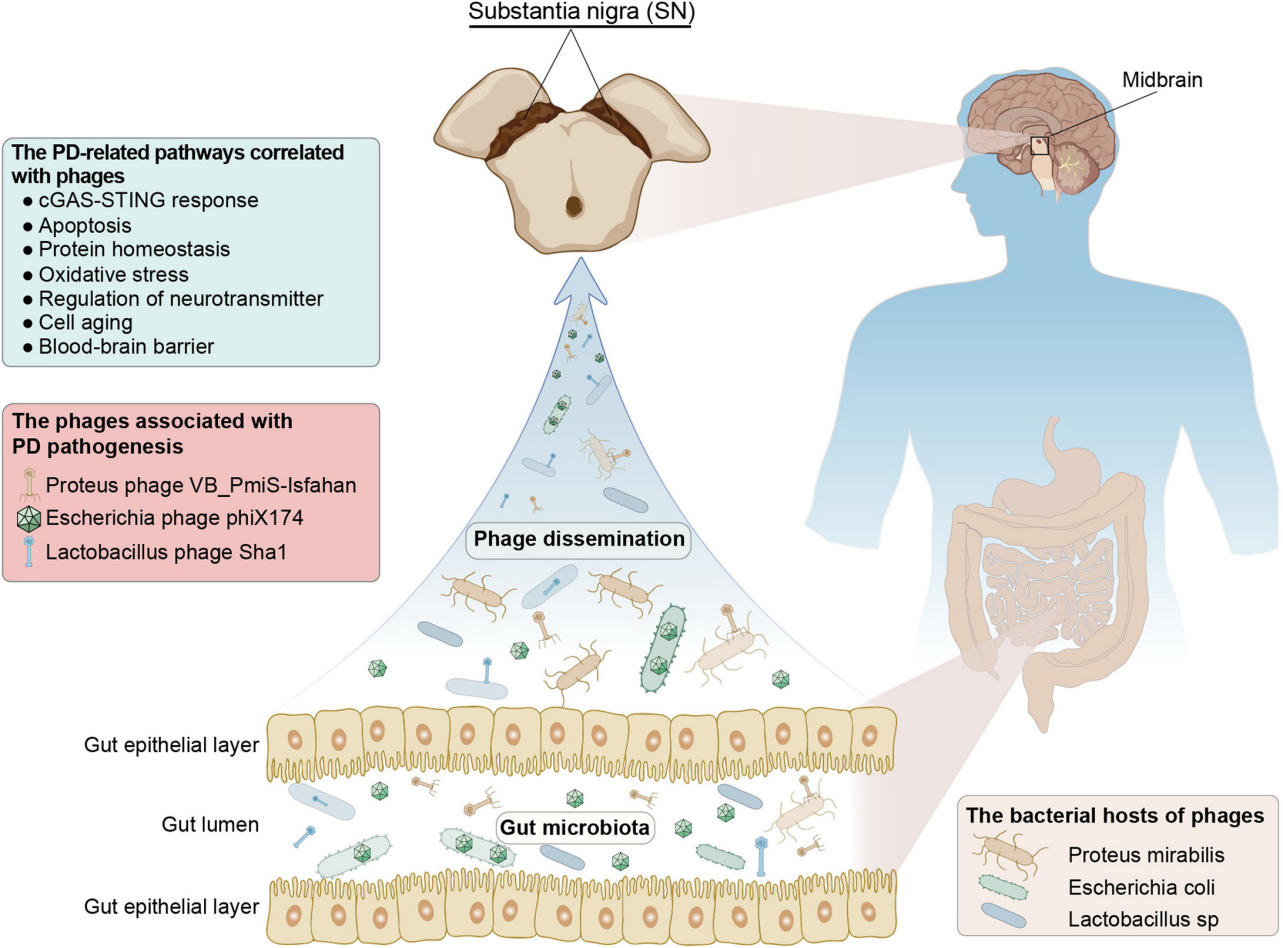
Schematic summary of hypothesized mechanism underlying the virobiota, phages, gut microbiota, and PD pathogenesis. The discovery of symbiotic virobiota in the SN. A symbiotic phages and the dysbiosis of virobiota in the SN may underlie PD pathogenesis. The phages may be one of the bridges between gut microbiota and PD pathogenesis.

Differentially expressed genes (DEGs) reflect molecular signatures of PD. A total of 151 DEGs were identified (Fig. 7A and Table S9). These DEGs were enriched for dopamine biosynthesis, cGAS-STING pathway, and response to the virus (Fig. 7B to D). PPI analysis of DEGs revealed similar results with VPGs (Fig. S4C and D). Receiver operating characteristic (ROC) curve analysis showed that these DEGs can differentiate PD patients from non-PD patients (Fig. S4E and F), demonstrating that cGAS-STING and antiviral systems may be involved in PD pathogenesis. Venn diagram also highlighted the importance of dopamine biosynthesis and cGAS-STING system in PD etiology (Fig. 8). Overall, similar to VPGs, DEGs uncovered a suppressed antiviral cGAS-STING pathway in the SN of PD patients, validating for the first time that symbiotic virobiota underlies PD pathogenesis, and provided a novel insight into the understanding of PD pathogenesis from the perspective of virus–human symbiosis (Fig 9).

### Intra-SNc viral challenge pathologically affects neural cells

To experimentally test whether the virus could affect the substantia nigra pars compacta (SNc) neural cells, and whether treatment with the virus may cause PD-related pathological processes, we injected herpes simplex virus-1 (HSV-1) into bilateral SNc of the mice and assessed viral and PD-related biochemical markers at day 7 after injection. The experimental procedure was described in Fig. S5A. The expression of infected-cell polypeptide 4 (ICP 4), a transcriptional regulatory protein that is essential for gene transcription of the virus, was detected during this acute phase of treatment. Notably, we found that the virus can infect dopaminergic neurons, astrocytes, and microglia (Fig. S5B to D). A reduced TH immunoreactivity, a loss of dopaminergic neurons, and an increased neuroinflammatory morphology of reactive astrocytes and microglia were observed, as compared with those of phosphate-buffered saline (PBS)-treated mice (Figs. S6, 7A and B, and 8A and B). Of note, the expression of ICP4, decreased TH^+^ fiber density, and reduced TH immunoreactivity were also detected in the striatum (Fig. S9A to D). Taken together, these results validated that the virus may affect SNc neural cells and the nigrostriatal pathway, suggesting that these may be linked to PD-related pathogenesis.

### Intra-SNc viral challenge induces parkinsonism

To experimentally examine whether the virus could induce parkinsonism, we evaluated PD-related biochemical markers and behaviors in month 5 after intra-SNc viral injection. The experimental procedure was described in Fig. S10A. We found a reduced TH immunoreactivity, a loss of dopaminergic neurons, and an increased number of astrocytes and microglia in the SNc, compared with that of the PBS-treated group (Figs. S10B to D and 11A and B). Decreased TH^+^ fiber density and reduced TH immunoreactivity were detected in the striatum treated with the virus (Fig. S12A to C). Pole descent, rotarod test, beam traversal, hindlimb clasping reflexes, and gait test revealed that viral challenge may lead to impaired motor coordination and balance (Fig. S13). Taken together, these results suggest that intra-SNc viral challenge may cause PD-related molecular and behavioral phenotypes.

## Discussion

For a century, the association between viruses and PD pathogenesis has been debated, but largely ignored or dismissed as controversial [30]. The link between viruses and PD has been defined as one of the medical mysteries, because clinical studies have shown that there was no history of an overt episode of viral infection in the vast majority of PD patients [28,29]. In 2022, viruses were observed in the amygdala, median temporal gyrus, SN, intestine, and blood of PD patients, showing that the positive rates of viruses in PD patients might be higher than those in non-PD individuals [75]. New concepts of “Virobiota” [44] and “Virome” [76] are put forward, revealing a new landscape of virus–human symbiosis, suggesting a potential association between symbiotic virobiota and PD pathogenesis. Nevertheless, the limitation of human brain biopsy and the difficulty for electron microscope in observing virions have been the major hurdles for deciphering whether or not viruses exist in human SN [77].

In this study, using worldwide RNA-seq datasets of the SN of PD patients [78–82], we observed the existence of viruses/virobiota in the human SN. We unveiled a dysbiosis in the SN virobiota of PD patients. We found that the phages that host the gut microbiota may be predominant in the SN virobiota of PD patients (Fig. 6). These observations suggest that virobiota may exert a lifelong influence on neural cells and finally may cause the loss of dopaminergic neurons in the SN. The phages that host the gut microbiota may enter the SN to live in symbiosis with dopaminergic neurons. The phages may be the main bridge between intestinal flora and PD pathogenesis. Together, we show that the virobiota in the SN may underlie PD etiology (Fig. 9).

The *Proteus* phage VB_PmiS-Isfahan is a phage that infects *Proteus mirabilis* [83], which is one of the dominant human gut bacteria [84]. This phage is present in semen and may be associated with the male fertility [85]. This phage is one of the top abundant viral strains in nasopharyngeal specimens of non-COVID individuals [86], and it can also be detected in pan-cancer samples [87]. In this study, we found that the *Proteus* phage VB_PmiS-Isfahan was a common virus in the SN of PD patients from the United Kingdom, the Netherlands, and Switzerland. The human gene expressions dampened by the *Proteus* phage VB_PmiS-Isfahan were enriched for PD-related pathways including cGAS-STING response, dopamine metabolic process, oxidative stress, and apoptosis (Fig. 6). The *Escherichia* phage phiX174 is a phage that infects *Escherichia coli* [88], which is an indicator of normal intestinal flora of humans. In 1962, Fiers and Sinsheimer [89] identified the phage phiX174 as a single-stranded DNA virus. In 1967, Kornberg and colleagues [90] used the phage phiX174 as the first in vitro model to prove that the synthesized DNA produces all the features of the natural virus. As the first DNA-based genome, Frederick Sanger’s group sequenced the genome of the phage phiX174 in 1977 [91,92]. The phage phiX174 can induce humoral immune response [93], and thus, it has been used to evaluate humoral immune function [93–97]. The phage phiX174 genome can be integrated into human lymphocyte genome [98]. The *Lactobacillus* phage Sha1 is a phage that infects *Lactobacillus* [99], and it is increased in the gut virobiota of older adults with mild cognitive impairment [100]. In this study, we observed a strong negative correlation between VRFC of these phages and PD-related human gene expression in the SN of PD patients (Fig. S2). Together, these findings reveal the importance of studying the relationship between phages and etiology of human diseases such as PD.

Dysbiosis of virobiota is involved in the onset and progression of diseases [44,101,102]. Species of phages was decreased, whereas viral community dissimilarity was higher in the gut virome of patients with ulcerative colitis than controls [103,104]. There is a difference in the gut virome between obese patients with type 2 diabetes mellitus and healthy controls [101]. In this study, we found that virome composition in the SN of PD patients and non-PD individuals was separated into 2 distinct clusters. The viral community dissimilarity among PD patients was higher than controls. The core species of viruses was higher, whereas unique species of viruses was lowered in the SN of PD patients compared with controls (Fig. 4). Overall, these results suggest that dysbiosis of virobiota in the SN may be involved in PD pathogenesis.

*Cnaphalocrocis medinalis* granulovirus is the most prevalent virus in the gut virome of patients with hypertension, whereas this virus is not a contributor to the hypertension [102]. Similarly, in this study, we found that the abundance of the *Acinetobacter* phage AbKT21phiIII and the *Escherichia* phage ESSI2_ev239 was enriched, whereas the abundance of the *Escherichia* phage Lambda_ev017 was diminished in virobiota of the SN of PD patients, and the viral gene expression of these phages was not correlated with PD-related human gene expression. These observations suggest that the abundance of symbiotic viruses in the SN may not be a hallmark of PD pathogenesis, reflecting a complex etiologic connection between symbiotic virobiota and human diseases.

Phages may cause neuroimmune responses of eukaryotic cells. A phage cocktail stimulates interferon-γ (IFN-γ) production in dendritic cells [105]. Staphylococcus phage reduces lipopolysaccharide-induced high levels of interleukin-1β (IL-1β) and IL-6 in mammary alveolar epithelial cells [106]. Phage lysates up-regulate IL-1β and IL-6 in peripheral blood mononuclear cells [107]. In this study, we found that VRFCs of the *Proteus* phage VB_PmiS-Isfahan, the *Escherichia* phage phiX174, and the *Lactobacillus* phage Sha1 were negatively correlated with the gene expression of proinflammatory cytokines, cGAS-STING pathway, and antiviral immunity. Together, these findings suggest that symbiotic phages in the SN may cause neuroinflammation and disrupt antiviral immune responses, which may contribute to PD pathogenesis.

The cGAS-STING system acts as a sensor for cytosol viral DNA upon viral infection and phage invasion [105,108–112]. Activation of cGAS-STING initiates anti-phage immune response to restrict phage activity [113–121]. The virus can inhibit the DNA-sensing function of the cGAS-STING system in humans [122]. In this study, we found that both VPGs and DEGs were enriched for cGAS-STING response, and the gene expression level of the cGAS-STING system was lowered in the SN of PD patients (Figs. 6 to 8). The cGAS-STING system gene expression was negatively correlated with phage gene expression in the SN of PD patients. Together, these findings suggest that cGAS-STING activity may be inhibited by symbiotic phages in the SN of PD patients, revealing a phage-suppressed cGAS-STING function in PD pathogenesis. The relief of phage-inhibited cGAS-STING activity may provide a promising strategy for prevention or treatment of PD.

Epidemiological evidence shows that viral infection may precede the appearance of PD symptoms [123]. In this study, the SN samples were from the PD patients aged between 65 and 90 years. Considering the potential symbiosis, we reasoned that the virobiota–neural cell symbiosis in the SN may predate the PD onset.

Amantadine, an anti-Parkinson agent [124], also serves as an antiviral medication. Amantadine can inhibit phage assembly [125]. Our previous study suggests that amantadine plays an antiviral role by activating the cGAS-STING pathway [126]. These findings raise the possibility that amantadine may eliminate the symbiotic phages to relieve the death of dopaminergic neurons in the SN.

Phages and eukaryotic viruses can enter neural cells to cause neuroinflammation or neuronal death. Bacteriophage 933W particles enter the brain limbic system to activate astrocytes and result in the death of the motor cortex neurons [63]. Intracerebral inoculation of Japanese encephalitis virus (JEV) induces the loss of SN dopaminergic neurons in rats [127]. The rectally administered bacteriophages can cross the BBB and activate neural cells to trigger the neuroinflammation in mice [52]. These studies suggest that the virobiota are implicated in the induction of the loss of SN dopaminergic neurons.

Next-generation sequencing technology can probe viral mRNA fragments of intracellular viruses or viral episomes. RNA-seq can detect virobiota in postmortem SN tissues. In this study, sequencing read length ranged from 50 to 80 base pairs. The contigs longer than 50 nucleotides showing at least 90% identity to reference viral genome were retained. This strategy guarantees high accuracy and sensitivity of virobiota annotation.

Viral infection may increase BBB permeability. BBB does not constitute a barrier to phages [52–54,128]. The filamentous phage M13 can cross BBB to access the brain after intranasal administration in mice [49,51,129]. Phages can be delivered into the brain through entering peripheral immune cells by the “Trojan horse mechanism” [48,130]. In this study, we found that gene expression of the phages was negatively correlated with BBB-related gene expression (Fig. S2). Together, we suggest that the phages may cross the BBB to symbiose with the SN.

Phage-derived antimicrobials have been broadly applied in clinical treatment, food industry, and aquaculture [131–133]. This strategy has been used to treat intestinal, skin, urinary, and respiratory infections [134,135]. Thus, to test whether phage-related therapy may lead to increased risk for PD, further investigations are needed.

Human–gut virome variation is influenced by geographic regions [136]. Our findings revealed that virobiota composition in the human SN was geographically related. Therefore, we suggest that SN samples of PD patients from more countries or regions are needed for assessing association between virobiota and PD pathogenesis.

The visualization of phages in brain tissue has been proven to be difficult due to the limitation of anti-phage antibodies. Thus, it is challenging to delineate the relationship between phages and neurodegenerative diseases by means of intracerebral injection of phages. As a routine neurotropic viral model, HSV-1 is often used to experimentally test the relationship between the virus and neurodegenerative diseases [137–145]. Therefore, in this study, by using stereotaxic injection of the virus into the SNc, we attempted to observe the influence of the viral existence in the SNc. We found that the virus could infect SNc dopaminergic neurons, astrocytes, and microglia and the nigrostriatal pathway. Moreover, the intra-SNc viral challenge caused PD-related molecular and behavioral phenotypes. Together, these observations validated that the virus could cause parkinsonism, and also implied that the phages, the most abundant types of viruses in the biosphere, might be linked to the PD pathogenesis.

In summary, this is the first study to discover virobiota or phagebiota in the SN. A lifelong low viral load of symbiotic virobiota in the SN may be a contributor to PD pathogenesis. The phages that host gut microbiota may be implicated in PD etiology. Our observations unlocked the black box between phages and PD, pointing out a complex etiologic connection between symbiotic virobiota and human diseases, providing a novel insight into PD etiology from the perspective of phage–human symbiosis. The further study of virobiota in the brain may shed light on PD pathogenesis and therapy.

## Methods

### Prevention of microbial RNA and ribonuclease contamination

To prevent microbial RNA or RNA enzyme contamination, sterile procedures for the SN dissection and collection were performed. For detailed information on the procedures of the SN dissection and collection, please refer to the brain bank websites: Netherlands Brain Bank (https://www.brainbank.nl/brain-tissue/autopsy/), the Parkinson’s UK Brain Bank (https://www.parkinsons.org.uk/research/parkinsons-uk-brain-bank), and the Geneva University Hospitals (https://www.hug.ch/en/clinical-pathology).

### Bioinformatic analysis

#### Data collection

We used “Parkinson’s disease” as keywords to search for genome-wide expression studies in the NCBI-GEO (http://www.ncbi.nlm.nih.gov/geo/) and European Genome-phenome Archive (EGA) platform (https://ega-archive.org/). The inclusion criteria included the following:

1. The studies that were designed for exploring gene expression in the SN for PD patients and non-PD individuals were the first choice for inclusion.
2. The study type was Gene Expression Profiling by RNA-seq or microarray.
3. The microarray studies comprised cell intensity file (CEL) raw files. Besides, to reduce the bias from different microarray platform, only data from 2 widely used platforms Affymetrix Human Genome U133A and Affymetrix Human Genome U133 Plus 2.0 were considered.

The raw RNA-seq data were retrieved from NCBI SRA database (https://www.ncbi.nlm.nih.gov/sra) or generously shared by P. Lingor and L. C. Gomes on the EGA platform.

We then performed analysis of 5 RNA-seq datasets (EGAD00001006883, GSE169755, GSE114918, GSE136666, and GSE114517) and 5 microarray datasets (GSE20141, GSE49036, GSE7621, GSE8397, and GSE20292). Details of the datasets are provided in Table S1. The information of PD patients and non-PD individuals in this study are provided in Table S2.

#### Analysis of virome composition and structure in the SN

To identify viral fragments from the RNA-seq data of the SN, the raw FASTQ sequencing reads were first preprocessed by fastp software (version 0.21.0, default parameters) [146] for quality control and adaptor trimming. Then, the reads were aligned to a merged reference genome file combining human reference genome (hg38) and a comprehensive collection of 13,559 virus genomes from viruSITE database (http://www.virusite.org/index.php, version 2021.2) [66] by using the STAR software (version 2.7.8) [147]. When running the STAR software, we adopted the parameters recommended by Viral-Track [64], which is a recently established computational pipeline for detecting viral reads from sequencing data. Based on the reads aligned to viral genome, viral read counts were obtained via Viral-Track to assess the abundance of each virus, i.e., VRFC. Instead of the built-in thresholds of Viral-Track, which were designed for the near full-length viral gene detection purpose [64], 2 alternative criteria were applied for false-positive control for our purpose of viral fragment detection:

1. Viral reads of each detected virus should be able to assemble short viral contigs. For each sample, all reads aligned to a virus genome were extracted by SAMtools (version 1.12) [148] and submitted to the Trinity contig assembly pipeline (version 2.13.2) [149] using the virus genome as the contig assembly reference and allowing no intron inside the contigs. Only assembled contigs longer than 50 nucleotides and showing at least 90% identity to the reference genome were retained.
2. All viral reads considered in virus quantification should not be aligned to any chromosome of the human refence genome.

To further assess the composition and structure of the SN virome, the viral read counts or the normalized viral RPKM (reads per kilobase per million reads mapped) were imported into R (version 4.0.2), as per the requirement of the software used in the subsequent analysis. Virus abundance, alpha diversity, and beta diversity were calculated with R package phyloseq (version 1.32.0) and vegan (version 2.6.4). Permutational multivariate analysis of variance (PERMANOVA) implemented in vegan package was performed for Bray–Curtis dissimilarity. Data visualization was performed by R packages ggplot2 (version 3.4.0) and aPCoA (version 1.3). The statistically significant differences between PD and non-PD groups were determined by 2-tailed Wilcoxon test using ggpubr (version 0.4.0) and ggsignif (version 0.6.3) R packages, and a *P* value of <0.05 was considered statistically significant.

#### Human gene expression quantification and its correlation with viral gene expression

Raw FASTQ reads were preprocessed and aligned to human genome using the same method as above. Then, based on the reads aligned to the known genes in human reference genome, the human gene expressions were quantified by the featureCounts method of Rsubread R package (version 2.4.3) [150], using the standard Ensembl gene annotation reference (http://www.ensembl.org/, version 104) and default parameters. Pearson’s correlation between virus expression and human gene expression in PD and non-PD patients was calculated using the cor.test function in R. We focused on the correlations regarding PD-related pathological genes. The PD-related human genes correlated to VRFC were termed VPGs. The correlation heatmap was generated using the pheatmap R package (version 1.0.12). The paired box plot was generated by the ggpubr R package (version 0.4.0), and the Wilcoxon signed-rank test was implied by the ggsignif R package (version 0.6.4).

#### Differential expression analysis and functional enrichment analysis

Both RNA-seq and microarray-based gene expression profiles were considered in the differential expression analysis. To be scalable to the microarray data, the gene expression values from RNA-seq data were firstly transformed to log_2_(*x* + 1). As for the microarray data, the raw CEL files were processed using the robust multichip average (RMA) method for background correction and normalization, which is implemented in the affy (version 1.68.0) and gcrma (version 2.62.0) R packages. After removing duplicated gene probes and unspecific probes, all probes were mapped to single Entrez Gene IDs according to the corresponding probe annotation files. Data from RNA-seq and microarray datasets were merged based on their shared genes, resulting in a gene expression matrix covering 12,180 genes. Batch effects were supervised by principal components analysis (PCA) method and removed using the ComBat function of the sva R package (version 3.38.0). Negative expression values introduced during batch effect removal were truncated to zero. Differential gene expression analysis was carried out by the limma R package (version 3.46.0), and genes with a *P* value of <0.05 and |log_2_ (Fold change) | > 1 were considered significant DEGs. Gene Ontology (GO) and Kyoto Encyclopedia of Genes and Genomes (KEGG) analyses for these DEGs were based on the Metascape database (http://metascape.org/gp/index.html#/main/step1), and a functional term with corrected *P* value < 0.05 was considered significantly enriched. The expression heatmap of all DEGs was plotted using pheatmap R package (version 1.0.12). The GO biological process network of DEGs was carried out by BiNGO [151] tool in Cytoscape software [152].

The STRING 11.5 database was used to predict the interactions of DEGs and virus-related genes identified in the study and to map the PPI network [153]. We selected the data analysis mode and default PPI confidence threshold of the STRING database to construct the PPI network. The protein networks were visualized by Cytoscape software [152] and analyzed by the Network Analyzer tool based on degree. The degree indicates the number of interactions of each protein. We compared the relative expression of the DEGs across 5 RNA-seq datasets in this study. To assess the diagnostic value of DEGs, we compared the expression of the hub node genes (i.e., genes with interactions > 5). A ROC curve was performed, and the ROC curve for each hub gene and their combination were calculated to screen for a better diagnostic potential.

### Mice and animal care

Male C57BL/6 mice were procured from Charles River Laboratories Beijing Branch (operating as Beijing Vital River Laboratory Animal Technology Co. Ltd.) and the Department of Laboratory Animal Science at the Peking University Health Science Center. All experimental protocols involving these mice were duly reviewed and received approval from the Institutional Care and Use Committee of the Peking University Health Science Center, under approval number LA2019340. The mice were maintained in a controlled environment at a temperature of 22 ± 1 °C, following a 12-h light/dark cycle (lights on from 20:00 to 08:00). Mice had ad libitum access to food and water throughout the study.

### Viral treatment

Mice were anesthetized using isoflurane and securely positioned within a stereotaxic frame. A small incision in the scalp was made to reveal the skull, preparing for precise brain interventions. Using a stereotaxic holder, the brains were stabilized to ensure accurate targeting. An injection was performed with a 0.2-mm stainless steel needle attached to a 5-μl Hamilton syringe, administering 2.5 μl of either an HSV-1 suspension (totaling approximately 1 × 10^3^ plaque-forming units) or PBS (as a control). The virus or PBS was bilaterally injected into the SNc, with specific coordinates from the Paxinos and Franklin brain atlas: anterior–posterior at −3.2 mm, dorsal–ventral at ±1.2 mm, and lateral at −4.6 mm. Morbidity and mortality were monitored twice a day. Neurological assessment was based on a graded scoring system from 1 to 5, designed to describe progressive neurological impairment: 1 signifies ruffled fur and hunched posture but can easily be made to move around; 2 indicates a hunched posture and slow to move; 3 describes a hunched posture, some movement, and labored breathing; 4 describes a hunched posture, labored breathing, and little or no movement; and 5 represents moribund or dead [154]. In our study, score 3 was not reached.

### Immunofluorescence

Details regarding primary antibodies and dilutions are provided in Table S10. Brain sections of 30-μm thickness were systematically prepared. The preparation involved transcardial perfusion of mice followed by brain fixation in 4% paraformaldehyde (PFA) over 2 days. Subsequent to fixation, the brains were immersed in a 20 to 30% sucrose gradient for cryoprotection and then embedded in OCT compound (Sakura FineTech, Tokyo). Brain sections were blocked using 10% goat serum in PBS containing 0.2% Triton X-100 and then incubated overnight at 4 °C with tyrosine hydroxylase (TH) or infected-cell polypeptide 4 (ICP 4) antibodies. Following primary incubation, sections were washed 3 times with PBS and incubated with fluorescently labeled secondary antibodies (Alexa Fluor 488 or 594, YEASEN, 1:400) for 2 h at room temperature. Imaging was performed using the Olympus VS120 Slide Scanning System. Analysis of TH-positive neurons in the SNc and the density of TH-positive fibers in the striatum was carried out using ImageJ software.

### Protein extraction and western analysis

Protein samples were harvested from the SNc or the striatum (STR) using radioimmunoprecipitation assay (RIPA) buffer composed of 0.5% NP-40, 0.1% sodium deoxycholate, 150 mM NaCl, and 50 mM tris-HCl (pH 7.4), along with added phosphatase (B15002, Bimake) and protease inhibitors (B14002, Bimake). The homogenates were then centrifuged at 12,000*g* for 30 min at 4 °C, and the supernatants were retained as protein extracts. Protein concentrations were determined using the bicinchoninic acid (BCA) assay method (Aidlab; PP01). Protein samples were mixed with a loading buffer containing 62.5 mM tris-Cl (pH 6.8), 2% SDS, 5% glycerol, and 0.05% bromophenol blue and then denatured at 95 °C for 5 min. Proteins were electrophoresed on a 10% sodium dodecyl sulfate–polyacrylamide gel electrophoresis (SDS-PAGE) and subsequently transferred onto a nitrocellulose membrane (Pall Corporation; T60327). The membranes were blocked using 5% skim milk in tris-buffered saline with Tween 20 (TBST) for 2 h at room temperature and incubated overnight at 4 °C with primary antibodies diluted in 5% bovine serum albumin (BSA)–TBST. Following primary incubation, membranes were washed thrice in TBST and incubated with horseradish peroxidase-conjugated secondary antibodies in 5% milk–TBST for 2 h at room temperature. After washing thrice for 15 min each in TBST, protein bands were visualized using an Enhanced Chemiluminescence system (Bio-Rad). The intensity of protein bands was quantified using ImageJ software (National Institutes of Health), and all original blot images are provided in Fig. S14.

### Behavioral tests

Motor functions in mice were evaluated using beam traversal, pole test, rotarod test, hindlimb scoring, and gait test. Mice were allowed to acclimate to the testing environment for 1 h on the test day and also on the preceding day. Prior to the assessments, mice underwent a 3-day training period, with each session followed by a 10-min interval. All equipment was sanitized with 75% ethanol after each trial to ensure cleanliness.

#### Beam traversal test

Beam test was used to detect subtle deficits in motor skills and balance of mice. A 100-cm wooden beam consists of 4 segments of 0.25 m in length. Each segment was of thinner widths 3.5, 2.5, 1.5, and 0.5 cm, with 1-cm overhangs placed 1 cm below the surface of the beam. In the test, mice were placed on the widest segment as a loading platform, the narrowest segment placed into a dark goal box. Mice were made to traverse the beam in the same manner (cutoff time 30 s maximum). The test time from the start to the 90-cm point was recorded. Timing began when the animals placed their forelimbs onto the 2.5-cm segment and ended when one forelimb reached the 90-cm point.

#### Pole test

The pole test is conducted on a wooden rod (diameter 8 mm; height 80 cm), which was wrapped with bandage gauze. The rod was fixed in the middle of an empty cage. Mice were placed on the top of a wooden pole and facing downward. The test time until it descended to the base of the pole was recorded with a maximum duration of 30 s. When the mouse was not able to turn downward and instead dropped from the pole, the test time was taken as the slowest mouse to pass the pole. The pole test is used to assess rigidity.

#### Rotarod test

Rotarod test evaluates motor coordination and motor learning of mice. In the test, mice were placed on the accelerating rotarod cylinder. After pretraining at 4 rpm for 1 min, the speed was gradually increased from 4 to 40 rpm within 5 min and kept at 40 rpm for an additional 2 min. A trial ended if mice fell off the rungs or gripped the device and spun around for 2 consecutive revolutions without attempting to walk on the rungs. Time before falling was automatically recorded with a maximum duration of 5 min. Data are presented as the percentage of the third trials on the rotarod compared to the control.

#### Hindlimb scoring

Mice were gently lifted upward by the mid-section of the tail and observed over 5 to 10 s. Mice were assigned a score of 0, 1, 2, and 3 based on the extent to which the hindlimbs clasped inward. The mice that freely moved and extended their limbs outward were scored as 0. A score of 1 was recorded if the mice kept one hindlimb inward while restrained or showed partial inward clasping with both legs. A score of 2 was assigned when both legs were clasped inward for most of the observation period, but still exhibited some flexibility. If mice exhibited full hindlimb paralysis with immediate inward clasping and no flexibility, a score of 3 was assigned.

#### Gait test

The testing apparatus is constructed from a 3-mm-thick gray acrylic board and includes a runway with nonslippery white paper (10 cm wide, 60 cm long, 12 cm tall) and a dark goal box (16 cm wide, 10 cm long, 12 cm tall). During the first training day, mice were familiarized with the equipment for 2 min before having their front and back paws colored red and black using safe food dyes. Mice were then trained to run to the goal box. In the test, mice were required to run the runway within a maximum time of 60 s. The analysis of footprint patterns focused on 3 parameters (stride length, stride width, and overlap), with prints near the beginning and end disregarded due to the impact of acceleration or deceleration. Stride length was measured as the average distance between each forepaw and hindpaw footprint. Stride width was measured as the average distance between the right and left footprint of each forepaw and hindpaw. At least 4 values were measured in each trial for each parameter.

### Statistical analysis

Data are expressed as mean ± standard error of means (SEM). Representative morphological images were taken from at least 3 biologically independent experiments with similar results. Statistical significance was determined using Student’s *t* test. *P* values were indicated with *P < 0.05, **P < 0.01, or ***P < 0.001 on graphs. Sample sizes (*n*), statistical tests, and *P* values are indicated in each figure legend.

## Data Availability

The raw RNA-seq data in this study were retrieved from NCBI-GEO (http://www.ncbi.nlm.nih.gov/geo/) or generously shared by P. Lingor and L. C. Gomes on the EGA platform (https://ega-archive.org/) (corresponding accession numbers: EGAD00001006883, GSE169755, GSE114918, GSE136666, and GSE114517). The raw microarray datasets in this study were retrieved from NCBI-GEO (corresponding accession numbers: GSE20141, GSE49036, GSE7621, GSE8397, and GSE20292). Details of the datasets are provided in Table S1. The information of PD patients and non-PD individuals in this study are provided in Table S2.

## References

[1] Homayoun H. Parkinson disease. Ann Intern Med. 2018;169(5):ITC33–ITC48. 30178019 10.7326/AITC201809040

[2] Gardoni A, Agosta F, Sarasso E, Basaia S, Canu E, Leocadi M, Castelnovo V, Tettamanti A, Volontè MA, Filippi M. Cerebellar alterations in Parkinson’s disease with postural instability and gait disorders. J Neurol. 2023;270(3):1735–1744. 36534200 10.1007/s00415-022-11531-y

[3] Obeso JA, Rodriguez-Oroz MC, Goetz CG, Marin C, Kordower JH, Rodriguez M, Hirsch EC, Farrer M, Schapira AHV, Halliday G. Missing pieces in the Parkinson’s disease puzzle. Nat Med. 2010;16(6):653–661. 20495568 10.1038/nm.2165

[4] Marogianni C, Sokratous M, Dardiotis E, Hadjigeorgiou GM, Bogdanos D, Xiromerisiou G. Neurodegeneration and inflammation—An interesting interplay in Parkinson’s disease. Int J Mol Sci. 2020;21(22):8421. 33182554 10.3390/ijms21228421 PMC7697354

[5] Brandebura AN, Paumier A, Onur TS, Allen NJ. Astrocyte contribution to dysfunction, risk and progression in neurodegenerative disorders. Nat Rev Neurosci. 2023;24(1):23–39. 36316501 10.1038/s41583-022-00641-1 PMC10198620

[6] Bartels T, De Schepper S, Hong S. Microglia modulate neurodegeneration in Alzheimer’s and Parkinson’s diseases. Science. 2020;370(6512):66–69. 33004513 10.1126/science.abb8587

[7] Ransohoff RM. How neuroinflammation contributes to neurodegeneration. Science. 2016;353(6301):777–783. 27540165 10.1126/science.aag2590

[8] Han D, Zheng W, Wang X, Chen Z. Proteostasis of α-synuclein and its role in the pathogenesis of Parkinson’s disease. Front Cell Neurosci. 2020;14:45. 32210767 10.3389/fncel.2020.00045 PMC7075857

[9] Calo L, Wegrzynowicz M, Santivañez-Perez J, Grazia M. Synaptic failure and alpha-synuclein. Mov Disord. 2016;31(2):169–177. 26790375 10.1002/mds.26479

[10] Rey NL, Steiner JA, Maroof N, Luk KC, Madaj Z, Trojanowski JQ, Lee VMY, Brundin P. Widespread transneuronal propagation of α-synucleinopathy triggered in olfactory bulb mimics prodromal Parkinson’s disease. J Exp Med. 2016;213(9):1759–1778. 27503075 10.1084/jem.20160368 PMC4995088

[11] Goedert M. NEURODEGENERATION. Alzheimer’s and Parkinson’s diseases: The prion concept in relation to assembled Aβ, tau, and α-synuclein. Science. 2015;349(6248):1255555. 26250687 10.1126/science.1255555

[12] Gubellini P, Picconi B, Di Filippo M, Calabresi P. Downstream mechanisms triggered by mitochondrial dysfunction in the basal ganglia: From experimental models to neurodegenerative diseases. Biochim Biophys Acta. 2010;1802(1):151–161. 19683569 10.1016/j.bbadis.2009.08.001

[13] Billingsley KJ, Barbosa IA, Bandrés-Ciga S, Quinn JP, Bubb VJ, Deshpande C, Botia JA, Reynolds RH, Zhang D, Simpson MA, et al. Mitochondria function associated genes contribute to Parkinson’s disease risk and later age at onset. NPJ Parkinsons Dis. 2019;5:8. 31123700 10.1038/s41531-019-0080-x PMC6531455

[14] Wegrzynowicz M, Bar-On D, Calo’ L, Anichtchik O, Iovino M, Xia J, Ryazanov S, Leonov A, Giese A, Dalley JW, et al. Depopulation of dense alpha-synuclein aggregates is associated with rescue of dopamine neuron dysfunction and death in a new Parkinson’s disease model. Acta Neuropathol. 2019;138(4):575–595. 31165254 10.1007/s00401-019-02023-x PMC6778064

[15] Area-Gomez E, Guardia-Laguarta C, Schon EA, Przedborski S. Mitochondria, OxPhos, and neurodegeneration: Cells are not just running out of gas. J Clin Invest. 2019;129(1):34–45. 30601141 10.1172/JCI120848 PMC6307938

[16] Mattson MP, Arumugam TV. Hallmarks of brain aging: Adaptive and pathological modification by metabolic states. Cell Metab. 2018;27(6):1176–1199. 29874566 10.1016/j.cmet.2018.05.011 PMC6039826

[17] Smolders S, Van Broeckhoven C. Genetic perspective on the synergistic connection between vesicular transport, lysosomal and mitochondrial pathways associated with Parkinson’s disease pathogenesis. Acta Neuropathol Commun. 2020;8(1):63. 32375870 10.1186/s40478-020-00935-4 PMC7201634

[18] Dawson TM, Dawson VL. Molecular pathways of neurodegeneration in Parkinson’s disease. Science. 2003;302(5646):819–822. 14593166 10.1126/science.1087753

[19] Soldner F, Stelzer Y, Shivalila CS, Abraham BJ, Latourelle JC, Barrasa MI, Goldmann J, Myers RH, Young RA, Jaenisch R. Parkinson-associated risk variant in distal enhancer of α-synuclein modulates target gene expression. Nature. 2016;533(7601):95–99. 27096366 10.1038/nature17939 PMC5042324

[20] Frydas A, Wauters E, van der Zee J, Van Broeckhoven C. Uncovering the impact of noncoding variants in neurodegenerative brain diseases. Trends Genet. 2022;38(3):258–272. 34535299 10.1016/j.tig.2021.08.010

[21] Borie C, Gasparini F, Verpillat P, Bonnet AM, Agid Y, Hetet G, Brice A, Dürr A, Grandchamp B, French Parkinson’s disease genetic study group. Association study between iron-related genes polymorphisms and Parkinson’s disease. J Neurol. 2002;249(7):801–804. 12140659 10.1007/s00415-002-0704-6

[22] Ravenholt RT, Foege WH. 1918 influenza, encephalitis lethargica, parkinsonism. Lancet. 1982;2(8303):860–864. 6126720 10.1016/s0140-6736(82)90820-0

[23] Marizzoni M, Provasi S, Cattaneo A, Frisoni GB. Microbiota and neurodegenerative diseases. Curr Opin Neurol. 2017;30(6):630–638. 28906270 10.1097/WCO.0000000000000496

[24] Levine KS, Leonard HL, Blauwendraat C, Iwaki H, Johnson N, Bandres-Ciga S, Ferrucci L, Faghri F, Singleton AB, Nalls MA. Virus exposure and neurodegenerative disease risk across national biobanks. Neuron. 2023;111(7):1086–1093.e2. 36669485 10.1016/j.neuron.2022.12.029 PMC10079561

[25] Pavel A, Murray DK, Stoessl AJ. COVID-19 and selective vulnerability to Parkinson’s disease. Lancet Neurol. 2020;19(9):719. 10.1016/S1474-4422(20)30269-6 PMC7434474 32822628

[26] Nwabuobi L, Zhang C, Henchcliffe C, Shah H, Sarva H, Lee A, Kamel H. Characteristics and outcomes of Parkinson’s disease individuals hospitalized with COVID-19 in a New York City Hospital system. Mov Disord Clin Pract. 2021;8(7):1100–1106. 34541022 10.1002/mdc3.13309 PMC8441912

[27] Merello M, Bhatia KP, Obeso JA. SARS-CoV-2 and the risk of Parkinson’s disease: Facts and fantasy. Lancet Neurol. 2021;20(2):94–95. 33253627 10.1016/S1474-4422(20)30442-7 PMC7834123

[28] Obeso JA, Monje MHG, Matarazzo M. Major advances in Parkinson’s disease over the past two decades and future research directions. Lancet Neurol. 2022;21(12):1076–1079. 36402154 10.1016/S1474-4422(22)00448-3

[29] Leta V, Urso D, Batzu L, Lau YH, Mathew D, Boura I, Raeder V, Falup-Pecurariu C, van Wamelen D, Ray Chaudhuri K. Viruses, parkinsonism and Parkinson’s disease: The past, present and future. J Neural Transm. 2022;129(9):1119–1132. 36036863 10.1007/s00702-022-02536-y PMC9422946

[30] Cocoros NM, Svensson E, Szépligeti SK, Vestergaard SV, Szentkúti P, Thomsen RW, Borghammer P, Sørensen HT, Henderson VW. Long-term risk of Parkinson disease following influenza and other infections. JAMA Neurol. 2021;78(12):1461–1470. 34694344 10.1001/jamaneurol.2021.3895 PMC8546623

[31] Lin WY, Lin MS, Weng YH, Yeh TH, Lin YS, Fong PY, Wu YR, Lu CS, Chen RS, Huang YZ. Association of antiviral therapy with risk of Parkinson disease in patients with chronic hepatitis C virus infection. JAMA Neurol. 2019;76(9):1019–1027. 31168563 10.1001/jamaneurol.2019.1368 PMC6551582

[32] Lehrer S, Rheinstein PH. Vaccination reduces risk of Alzheimer’s disease, Parkinson’s disease and other neurodegenerative disorders. Discov Med. 2022;34(172):97–101. 36281030 PMC9608336

[33] Mwatelah R, McKinnon LR, Baxter C, Abdool Karim Q, Abdool Karim SS. Mechanisms of sexually transmitted *infection*-induced inflammation in women: Implications for HIV risk. J Int AIDS Soc. 2019;22(Suppl 6): Article e25346. 31468677 10.1002/jia2.25346 PMC6715949

[34] Ross AG, Olds GR, Cripps AW, Farrar JJ, McManus DP. Enteropathogens and chronic illness in returning travelers. N Engl J Med. 2013;368(19):1817–1825. 23656647 10.1056/NEJMra1207777

[35] Longo DL, Baden LR. Exploiting viruses to treat diseases. N Engl J Med. 2018;379(2):194–196. 29943655 10.1056/NEJMe1807181

[36] Ropper AH. Neurosyphilis. N Engl J Med. 2019;381(14):1358–1363. 31577877 10.1056/NEJMra1906228

[37] Sack J, Garcia-Tsao G. Variceal hemorrhage in a patient with hepatitis C virus cirrhosis in whom liver synthetic function had normalized after viral elimination. Hepatology. 2016;63(5):1733–1735. 26806550 10.1002/hep.28470 PMC4840037

[38] Wong G, Li S, Liu L, Liu Y, Bi Y. Zika virus in the testes: Should we be worried? Protein Cell. 2017;8(3):162–164. 28110372 10.1007/s13238-016-0357-3 PMC5326625

[39] Mateus AL, Otete HE, Beck CR, Dolan GP, Nguyen-Van-Tam JS. Effectiveness of travel restrictions in the rapid containment of human influenza: A systematic review. Bull World Health Organ. 2014;92(12):868–880D. 25552771 10.2471/BLT.14.135590 PMC4264390

[40] Birbeck GL. Zika virus: What the neurologist wants to know. Neurology. 2016;86(14):1272–1274. 26896047 10.1212/WNL.0000000000002553

[41] Siddiqi OK, Elafros MA, Bositis CM, Koralnik IJ, Theodore WH, Okulicz JF, Kalungwana L, Potchen MJ, Sikazwe I, Birbeck GL. New-onset seizure in HIV-infected adult Zambians: A search for causes and consequences. Neurology. 2017;88(5):477–482. 28003499 10.1212/WNL.0000000000003538 PMC5278945

[42] Maschke M, Kastrup O, Forsting M, Diener HC. Update on neuroimaging in infectious central nervous system disease. Curr Opin Neurol. 2004;17(4):475–480. 15247545 10.1097/01.wco.0000137540.29857.bf

[43] Sejvar JJ. West Nile virus infection. Microbiol Spectr. 2016;4(3). 10.1128/microbiolspec.EI10-0021-2016 27337465

[44] Duerkop BA, Hooper LV. Resident viruses and their interactions with the immune system. Nat Immunol. 2013;14(7):654–659. 23778792 10.1038/ni.2614 PMC3760236

[45] Bodner K, Melkonian AL, Covert MW. The enemy of my enemy: New insights regarding bacteriophage-mammalian cell interactions. Trends Microbiol. 2021;29(6):528–541. 33243546 10.1016/j.tim.2020.10.014

[46] Salmond GP, Fineran PC. A century of the phage: Past, present and future. Nat Rev Microbiol. 2015;13(12):777–786. 26548913 10.1038/nrmicro3564

[47] Popescu M, Van Belleghem JD, Khosravi A, Bollyky PL. Bacteriophages and the immune system. Annu Rev Virol. 2021;8(1):415–435. 34014761 10.1146/annurev-virology-091919-074551

[48] Barr JJ. A bacteriophages journey through the human body. Immunol Rev. 2017;279(1):106–122. 28856733 10.1111/imr.12565

[49] Dubos RJ, Straus JH, Pierce C. The multiplication of bacteriophage in vivo and its protective effect against an experimental infection with shigella dysenteriae. J Exp Med. 1943;78(3):161–168. 19871319 10.1084/jem.78.3.161 PMC2135327

[50] Keller R, Engley FB Jr. Fate of bacteriophage particles introduced into mice by various routes. Proc Soc Exp Biol Med. 1958;98(3):577–580. 13567777 10.3181/00379727-98-24112

[51] Frenkel D, Solomon B. Filamentous phage as vector-mediated antibody delivery to the brain. Proc Natl Acad Sci USA. 2002;99(8):5675–5679. 11960022 10.1073/pnas.072027199 PMC122830

[52] Podlacha M, Grabowski Ł, Kosznik-Kawśnicka K, Zdrojewska K, Stasiłojć M, Węgrzyn G, Węgrzyn A. Interactions of bacteriophages with animal and human organisms—Safety issues in the light of phage therapy. Int J Mol Sci. 2021;22(16):8937. 34445641 10.3390/ijms22168937 PMC8396182

[53] Møller-Olsen C, Ross T, Leppard KN, Foisor V, Smith C, Grammatopoulos DK, Sagona AP. Bacteriophage K1F targets *Escherichia coli* K1 in cerebral endothelial cells and influences the barrier function. Sci Rep. 2020;10(1):8903. 32483257 10.1038/s41598-020-65867-4 PMC7264188

[54] Jędrusiak A, Fortuna W, Majewska J, Górski A, Jończyk-Matysiak E. Phage interactions with the nervous system in health and disease. Cells. 2023;12(13):1720. 37443756 10.3390/cells12131720 PMC10341288

[55] Møller-Olsen C, Ho SFS, Shukla RD, Feher T, Sagona AP. Engineered K1F bacteriophages kill intracellular *Escherichia coli* K1 in human epithelial cells. Sci Rep. 2018;8(1):17559. 30510202 10.1038/s41598-018-35859-6 PMC6277420

[56] Nieth A, Verseux C, Barnert S, Süss R, Römer W. A first step toward liposome-mediated intracellular bacteriophage therapy. Expert Opin Drug Deliv. 2015;12(9):1411–1424. 25937143 10.1517/17425247.2015.1043125

[57] Øie CI, Wolfson DL, Yasunori T, Dumitriu G, Sørensen KK, McCourt P, Ahluwalia BS, Smedsrød B. Liver sinusoidal endothelial cells contribute to the uptake and degradation of entero bacterial viruses. Sci Rep. 2020;10(1):898. 31965000 10.1038/s41598-020-57652-0 PMC6972739

[58] Nguyen S, Baker K, Padman BS, Patwa R, Dunstan RA, Weston TA, Schlosser K, Bailey B, Lithgow T, Lazarou M, et al. Bacteriophage transcytosis provides a mechanism to cross epithelial cell layers. MBio. 2017;8(6):e01874-17. 29162715 10.1128/mBio.01874-17 PMC5698557

[59] Thyagarajan B, Olivares EC, Hollis RP, Ginsburg DS, Calos MP. Site-specific genomic integration in mammalian cells mediated by phage φC31 integrase. Mol Cell Biol. 2001;21(12):3926–3934. 11359900 10.1128/MCB.21.12.3926-3934.2001 PMC87055

[60] Merril CR, Geier MR, Petricciani JC. Bacterial virus gene expression in human cells. Nature. 1971;233(5319):398–400. 4940436 10.1038/233398a0

[61] Geier MR, Merril CR. Lambda phage transcription in human fibroblasts. Virology. 1972;47(3):638–643. 4551993 10.1016/0042-6822(72)90553-3

[62] Agu CA, Klein R, Lengler J, Schilcher F, Gregor W, Peterbauer T, Bläsi U, Salmons B, Günzburg WH, Hohenadl C. Bacteriophage-encoded toxins: The lambda-holin protein causes caspase-independent non-apoptotic cell death of eukaryotic cells. Cell Microbiol. 2007;9(7):1753–1765. 17346308 10.1111/j.1462-5822.2007.00911.x

[63] Del Cogliano ME, Pinto A, Goldstein J, Zotta E, Ochoa F, Fernández-Brando RJ, Muniesa M, Ghiringhelli PD, Palermo MS, Bentancor LV. Relevance of bacteriophage 933W in the development of hemolytic uremic syndrome (HUS). Front Microbiol. 2018;9:3104. 30619183 10.3389/fmicb.2018.03104 PMC6300567

[64] Bost P, Giladi A, Liu Y, Bendjelal Y, Xu G, David E, Blecher-Gonen R, Cohen M, Medaglia C, Li H, et al. Host-viral infection maps reveal signatures of severe COVID-19 patients. Cell. 2020;181(7):1475–1488.e12. 32479746 10.1016/j.cell.2020.05.006 PMC7205692

[65] Melnick M, Gonzales P, LaRocca TJ, Song Y, Wuu J, Benatar M, Oskarsson B, Petrucelli L, Dowell RD, Link CD, et al. Application of a bioinformatic pipeline to RNA-seq data identifies novel virus-like sequence in human blood. G3 (Bethesda). 2021;11(9):jkab141. 33914880 10.1093/g3journal/jkab141 PMC8661426

[66] Stano M, Beke G, Klucar L. viruSITE-integrated database for viral genomics. Database. 2016;2016:baw162. 28025349 10.1093/database/baw162 PMC5199161

[67] Wang Z, Hao Y, Zhang C, Wang Z, Liu X, Li G, Sun L, Liang J, Luo J, Zhou D, et al. The landscape of viral expression reveals clinically relevant viruses with potential capability of promoting malignancy in lower-grade glioma. Clin Cancer Res. 2017;23(9):2177–2185. 27864420 10.1158/1078-0432.CCR-16-1495

[68] Mathew D, Giles JR, Baxter AE, Oldridge DA, Greenplate AR, Wu JE, Alanio C, Kuri-Cervantes L, Pampena MB, D’Andrea K, et al. Deep immune profiling of COVID-19 patients reveals distinct immunotypes with therapeutic implications. Science. 2020;369(6508):eabc8511. 32669297 10.1126/science.abc8511 PMC7402624

[69] Zheng B, Liao Z, Locascio JJ, Lesniak KA, Roderick SS, Watt ML, Eklund AC, Zhang-James Y, Kim PD, Hauser MA, et al. PGC-1α, a potential therapeutic target for early intervention in Parkinson’s disease. Sci Transl Med. 2010;2(52):52ra73. 10.1126/scitranslmed.3001059 PMC3129986 20926834

[70] Dijkstra AA, Ingrassia A, de Menezes RX, van Kesteren R, Rozemuller AJ, Heutink P, van de Berg W. Evidence for immune response, axonal dysfunction and reduced endocytosis in the substantia nigra in early stage Parkinson’s disease. PLOS ONE. 2015;10(6): Article e0128651. 26087293 10.1371/journal.pone.0128651 PMC4472235

[71] Lesnick TG, Papapetropoulos S, Mash DC, Ffrench-Mullen J, Shehadeh L, de Andrade M, Henley JR, Rocca WA, Ahlskog JE, Maraganore DM. A genomic pathway approach to a complex disease: Axon guidance and Parkinson disease. PLOS Genet. 2007;3(6): Article e98. 17571925 10.1371/journal.pgen.0030098 PMC1904362

[72] Moran LB, Duke DC, Deprez M, Dexter DT, Pearce RK, Graeber MB. Whole genome expression profiling of the medial and lateral substantia nigra in Parkinson’s disease. Neurogenetics. 2006;7(1):1–11. 16344956 10.1007/s10048-005-0020-2

[73] Duke DC, Moran LB, Pearce RK, Graeber MB. The medial and lateral substantia nigra in Parkinson’s disease: mRNA profiles associated with higher brain tissue vulnerability. Neurogenetics. 2007;8(2):83–94. 17211632 10.1007/s10048-006-0077-6

[74] Zhang Y, James M, Middleton FA, Davis RL. Transcriptional analysis of multiple brain regions in Parkinson’s disease supports the involvement of specific protein processing, energy metabolism, and signaling pathways, and suggests novel disease mechanisms. Am J Med Genet B Neuropsychiatr Genet. 2005;137B(1):5–16. 15965975 10.1002/ajmg.b.30195

[75] Deng L, Fu P, Ding L, Duan X, Feng S, Peng Y. Virome analysis provides new insights into the association between viruses and Parkinson’s disease. J Med Virol. 2022;95(1):e28111. 36042689 10.1002/jmv.28111

[76] Anderson NG, Gerin JL, Anderson NL. Global screening for human viral pathogens. Emerg Infect Dis. 2003;9(7):768–774. 12890315 10.3201/eid0907.030004 PMC3023425

[77] Schwartz J, Elizan TS. Search for viral particles and virus-specific products in idiopathic Parkinson disease brain material. Ann Neurol. 1979;6(3):261–263. 534425 10.1002/ana.410060314

[78] Zaccaria A, Antinori P, Licker V, Kövari E, Lobrinus JA, Burkhard PR. Multiomic Analyses of Dopaminergic Neurons Isolated from Human Substantia Nigra in Parkinson’s Disease: A Descriptive and Exploratory Study. Cell Mol Neurobiol. 2022;42(8):2805–2818. 34528139 10.1007/s10571-021-01146-8 PMC9561004

[79] Aguila J, Cheng S, Kee N, Cao M, Wang M, Deng Q, Hedlund E. Spatial RNA Sequencing Identifies Robust Markers of Vulnerable and Resistant Human Midbrain Dopamine Neurons and Their Expression in Parkinson’s Disease. Front Mol Neurosci. 2021;14: Article 699562. 34305528 10.3389/fnmol.2021.699562 PMC8297217

[80] Xicoy H, Brouwers JF, Wieringa B, Martens GJM. Explorative Combined Lipid and Transcriptomic Profiling of Substantia Nigra and Putamen in Parkinson’s Disease. Cells. 2020;9(9):1966. 32858884 10.3390/cells9091966 PMC7564986

[81] Simchovitz A, Hanan M, Yayon N, Lee S, Bennett ER, Greenberg DS, Kadener S, Soreq H. A lncRNA survey finds increases in neuroprotective LINC-PINT in Parkinson's disease substantia nigra. Aging Cell. 2020;19(3): Article e13115. 32080970 10.1111/acel.13115 PMC7059180

[82] Caldi Gomes L, Galhoz A, Jain G, Roser AE, Maass F, Carboni E, Barski E, Lenz C, Lohmann K, Klein C, et al. Multi-omic landscaping of human midbrains identifies disease-relevant molecular targets and pathways in advanced-stage Parkinson’s disease. Clin Transl Med. 2022;12(1): Article e692. 35090094 10.1002/ctm2.692 PMC8797064

[83] Yazdi M, Bouzari M, Ghaemi EA. Genomic analyses of a novel bacteriophage (VB_PmiS-Isfahan) within Siphoviridae family infecting Proteus mirabilis. Genomics. 2019;111(6):1283–1291. 30149052 10.1016/j.ygeno.2018.08.008

[84] Yatsunenko T, Rey FE, Manary MJ, Trehan I, Dominguez-Bello MG, Contreras M, Magris M, Hidalgo G, Baldassano RN, Anokhin AP, et al. Human gut microbiome viewed across age and geography. Nature. 2012;486(7402):222–227. 22699611 10.1038/nature11053 PMC3376388

[85] Corral-Vazquez C, Blanco J, Aiese Cigliano R, Zaida S, Vidal F, Anton E. A transcriptomic insight into the human sperm microbiome through next-generation sequencing. Syst Biol Reprod Med. 2023;69(3):188–195. 36897835 10.1080/19396368.2023.2183912

[86] Hoque MN, Rahman MS, Ahmed R, Hossain MS, Islam MS, Islam T, Hossain MA, Siddiki AZ. Diversity and genomic determinants of the microbiomes associated with COVID-19 and non-COVID respiratory diseases. Gene Rep. 2021;23: Article 101200. 33977168 10.1016/j.genrep.2021.101200 PMC8102076

[87] Zhao L, Cho WCS, Luo JL. Exploring the patient-microbiome interaction patterns for pan-cancer. Comput Struct Biotechnol J. 2022;20:3068–3079. 35782745 10.1016/j.csbj.2022.06.012 PMC9233187

[88] Tessman I. Some unusual properties of the nucleic acid in bacteriophages S13 and phi X174. Virology. 1959;7(3):263–275. 13669296 10.1016/0042-6822(59)90197-7

[89] Fiers W, Sinsheimer RL. The structure of the DNA of bacteriophage phi-X174. III. Ultracentrifugal evidence for a ring structure. J Mol Biol. 1962;5:424–434. 13945085 10.1016/s0022-2836(62)80031-x

[90] Goulian M, Kornberg A, Sinsheimer RL. Enzymatic synthesis of DNA, XXIV. Synthesis of infectious phage phi-X174 DNA. Proc Natl Acad Sci USA. 1967;58(6):2321–2328. 4873588 10.1073/pnas.58.6.2321 PMC223838

[91] Sanger F, Air GM, Barrell BG, Brown NL, Coulson AR, Fiddes CA, Hutchison CA, Slocombe PM, Smith M. Nucleotide sequence of bacteriophage phi X174 DNA. Nature. 1977;265(5596):687–695. 870828 10.1038/265687a0

[92] Brussow H, Hendrix RW. Phage genomics: small is beautiful. Cell. 2002;108(1):13–16. 11792317 10.1016/s0092-8674(01)00637-7

[93] Pyun KH, Ochs HD, Wedgwood RJ, Yang XQ, Heller SR, Reimer CB. Human antibody responses to bacteriophage phi X 174: sequential induction of IgM and IgG subclass antibody. Clin Immunol Immunopathol. 1989;51(2):252–263. 2522846 10.1016/0090-1229(89)90024-x

[94] Ochs HD, Davis SD, Wedgwood RJ. Immunologic responses to bacteriophage phi-X 174 in immunodeficiency diseases. J Clin Invest. 1971;50(12):2559–2568. 5129308 10.1172/JCI106756 PMC292205

[95] Cunningham-Rundles C, Bodian C, Ochs HD, Martin S, Reiter-Wong M, Zhuo Z. Long-term low-dose IL-2 enhances immune function in common variable immunodeficiency. Clin Immunol. 2001;100(2):181–190. 11465947 10.1006/clim.2001.5052

[96] Bucknall R, Bacon P, Elson C, Jones JV. Antibody producing capacity to the bacteriophage phi X174 in rheumatoid arthritis. Ann Rheum Dis. 1987;46(12):889–897. 2962541 10.1136/ard.46.12.889 PMC1003418

[97] Uhr JW, Dancis J, Franklin EC, Finkelstein MS, Lewis EW. The antibody response to bacteriophage phi-X 174 in newborn premature infants. J Clin Invest. 1962;41(7):1509–1513. 13923602 10.1172/JCI104606 PMC291062

[98] Wenger SL, Steele MW, Turner JH. Incorporation of bacteriophage DNA into the genome of cultured human lymphocytes. In Vitro. 1981;17(8):695–700. 7327598 10.1007/BF02628405

[99] Yoon BH, Jang SH, Chang HI. Sequence analysis of the Lactobacillus temperate phage Sha1. Arch Virol. 2011;156(9):1681–1684. 21701917 10.1007/s00705-011-1048-2

[100] Chaudhari DS, Jain S, Yata VK, Mishra SP, Kumar A, Fraser A, Kociolek J, Dangiolo M, Smith A, Golden A, et al. Unique trans-kingdom microbiome structural and functional signatures predict cognitive decline in older adults. Geroscience. 2023;45(5):2819–2834. 37213047 10.1007/s11357-023-00799-1 PMC10643725

[101] Yang K, Niu J, Zuo T, Sun Y, Xu Z, Tang W, Liu Q, Zhang J, Ng EKW, Wong SKH, et al. Alterations in the Gut Virome in Obesity and Type 2 Diabetes Mellitus. Gastroenterology. 2021;161(4):1257–1269.e13. 34175280 10.1053/j.gastro.2021.06.056

[102] Han M, Yang P, Zhong C, Ning K. The Human Gut Virome in Hypertension. Front Microbiol. 2018;9:3150. 30619215 10.3389/fmicb.2018.03150 PMC6305721

[103] Manrique P, Bolduc B, Walk ST, van der Oost J, de Vos WM, Young MJ. Healthy human gut phageome. Proc Natl Acad Sci USA. 2016;113(37):10400–10405. 27573828 10.1073/pnas.1601060113 PMC5027468

[104] Zuo T, Lu XJ, Zhang Y, Cheung CP, Lam S, Zhang F, Tang W, Ching JYL, Zhao R, Chan PKS, et al. Gut mucosal virome alterations in ulcerative colitis. Gut. 2019;68(7):1169–1179. 30842211 10.1136/gutjnl-2018-318131 PMC6582748

[105] Gogokhia L, Buhrke K, Bell R, Hoffman B, Brown DG, Hanke-Gogokhia C, Ajami NJ, Wong MC, Ghazaryan A, Valentine JF, et al. Expansion of Bacteriophages Is Linked to Aggravated Intestinal Inflammation and Colitis. Cell Host Microbe. 2019;25(2):285–299.e8. 30763538 10.1016/j.chom.2019.01.008 PMC6885004

[106] Zhang L, Hou X, Sun L, He T, Wei R, Pang M, Wang R. Staphylococcus aureus Bacteriophage Suppresses LPS-Induced Inflammation in MAC-T Bovine Mammary Epithelial Cells. Front Microbiol. 2018;9:1614. 30083140 10.3389/fmicb.2018.01614 PMC6064726

[107] Van Belleghem JD, Clement F, Merabishvili M, Lavigne R, Vaneechoutte M. Pro- and anti-inflammatory responses of peripheral blood mononuclear cells induced by Staphylococcus aureus and Pseudomonas aeruginosa phages. Sci Rep. 2017;7(1):8004. 28808331 10.1038/s41598-017-08336-9 PMC5556114

[108] Sweere JM, Van Belleghem JD, Ishak H, Basch MS, Popescu M, Sunkari V, Kaber G, Manasherob R, Suh GA, Coa C, et al. Bacteriophage trigger antiviral immunity and prevent clearance of bacterial infection. Science. 2019;363(6434):eaat9691. 30923196 10.1126/science.aat9691 PMC6656896

[109] Zhou H, Tang YD, Zheng C. Revisiting IRF1-mediated antiviral innate immunity. Cytokine Growth Factor Rev. 2022;64:1–6. 35090813 10.1016/j.cytogfr.2022.01.004

[110] Franz KM, Neidermyer WJ, Tan YJ, Whelan SPJ, Kagan JC. STING-dependent translation inhibition restricts RNA virus replication. Proc Natl Acad Sci USA. 2018;115(9):E2058–E2067. 29440426 10.1073/pnas.1716937115 PMC5834695

[111] Cohen D, Melamed S, Millman A, Shulman G, Oppenheimer-Shaanan Y, Kacen A, Doron S, Amitai G, Sorek R. Cyclic GMP-AMP signalling protects bacteria against viral infection. Nature. 2019;574(7780):691–695. 31533127 10.1038/s41586-019-1605-5

[112] Hampton HG, Watson BNJ, Fineran PC. The arms race between bacteria and their phage foes. Nature. 2020;577(7790):327–336. 31942051 10.1038/s41586-019-1894-8

[113] Morehouse BR, Govande AA, Millman A, Keszei AFA, Lowey B, Ofir G, Shao S, Sorek R, Kranzusch PJ. STING cyclic dinucleotide sensing originated in bacteria. Nature. 2020;586(7829):429–433. 32877915 10.1038/s41586-020-2719-5 PMC7572726

[114] Sun L, Wu J, Du F, Chen X, Chen ZJ. Cyclic GMP-AMP synthase is a cytosolic DNA sensor that activates the type I interferon pathway. Science. 2013;339(6121):786–791. 23258413 10.1126/science.1232458 PMC3863629

[115] Margolis SR, Wilson SC, Vance RE. Evolutionary Origins of cGAS-STING Signaling. Trends Immunol. 2017;38(10):733–743. 28416447 10.1016/j.it.2017.03.004

[116] Duncan-Lowey B, Kranzusch PJ. CBASS phage defense and evolution of antiviral nucleotide signaling. Curr Opin Immunol. 2022;74:156–163. 35123147 10.1016/j.coi.2022.01.002

[117] Wein T, Sorek R. Bacterial origins of human cell-autonomous innate immune mechanisms. Nat Rev Immunol. 2022;22(10):629–638. 35396464 10.1038/s41577-022-00705-4

[118] Wu J, Sun L, Chen X, du F, Shi H, Chen C, Chen ZJ. Cyclic GMP-AMP is an endogenous second messenger in innate immune signaling by cytosolic DNA. Science. 2013;339(6121):826–830. 23258412 10.1126/science.1229963 PMC3855410

[119] Ishikawa H, Barber GN. STING is an endoplasmic reticulum adaptor that facilitates innate immune signalling. Nature. 2008;455(7213):674–678. 18724357 10.1038/nature07317 PMC2804933

[120] Ablasser A, Chen ZJ. cGAS in action: Expanding roles in immunity and inflammation. Science. 2019;363(6431):eaat8657. 30846571 10.1126/science.aat8657

[121] Jenson JM, Li T, Du F, Ea CK, Chen ZJ. Ubiquitin-like conjugation by bacterial cGAS enhances anti-phage defence. Nature. 2023;616(7956):326–331. 36848932 10.1038/s41586-023-05862-7 PMC10097602

[122] Webb LG, Fernandez-Sesma A. RNA viruses and the cGAS-STING pathway: reframing our understanding of innate immune sensing. Curr Opin Virol. 2022;53: Article 101206. 35180533 10.1016/j.coviro.2022.101206

[123] Smeyne RJ, Noyce AJ, Byrne M, Savica R, Marras C. Infection and Risk of Parkinson’s Disease. J Parkinsons Dis. 2021;11(1):31–43. 33361610 10.3233/JPD-202279 PMC7990414

[124] Perez-Lloret S, Rascol O. Efficacy and safety of amantadine for the treatment of L-DOPA-induced dyskinesia. J Neural Transm (Vienna). 2018;125(8):1237–1250. 29511826 10.1007/s00702-018-1869-1

[125] Choo SY, Vollherbst K, Keith A, Snipes W. Effects of adamantane derivatives on the stability and assembly of bacteriophage PM2. Can J Microbiol. 1982;28(7):897–900. 7172140 10.1139/m82-134

[126] Zhao M, Wang B, Zhang C, Su Z, Guo B, Zhao Y, Zheng R. The DJ1-Nrf2-STING axis mediates the neuroprotective effects of Withaferin A in Parkinson’s disease. Cell Death Differ. 2021;28(8):2517–2535. 33762743 10.1038/s41418-021-00767-2 PMC8329302

[127] Yadav P, Chakraborty P, Jha NK, Dewanjee S, Jha AK, Panda SP, Mishra PC, Dey A, Jha SK. Molecular Mechanism and Role of Japanese Encephalitis Virus Infection in Central Nervous System-Mediated Diseases. Viruses. 2022;14(12):2686. 36560690 10.3390/v14122686 PMC9781168

[128] Spindler KR, Hsu TH. Viral disruption of the blood-brain barrier. Trends Microbiol. 2012;20(6):282–290. 22564250 10.1016/j.tim.2012.03.009 PMC3367119

[129] Messing J. Phage M13 for the treatment of Alzheimer and Parkinson disease. Gene. 2016;583(2):85–89. 26869319 10.1016/j.gene.2016.02.005

[130] Lathe R, St CD. From conifers to cognition: Microbes, brain and behavior. Genes Brain Behav. 2020;19(8): Article e12680. 32515128 10.1111/gbb.12680

[131] Kakasis A, Panitsa G. Bacteriophage therapy as an alternative treatment for human infections. A comprehensive review. Int J Antimicrob Agents. 2019;53(1):16–21. 30236954 10.1016/j.ijantimicag.2018.09.004

[132] Vikram A, Callahan MT, Woolston JW, Sharma M, Sulakvelidze A. Phage biocontrol for reducing bacterial foodborne pathogens in produce and other foods. Curr Opin Biotechnol. 2022;78: Article 102805. 36162186 10.1016/j.copbio.2022.102805

[133] Ramos-Vivas J, Superio J, Galindo-Villegas J, Acosta F. Phage Therapy as a Focused Management Strategy in Aquaculture. Int J Mol Sci. 2021;22(19):10436. 34638776 10.3390/ijms221910436 PMC8508683

[134] Chang RYK, Nang SC, Chan HK, Li J. Novel antimicrobial agents for combating antibiotic-resistant bacteria. Adv Drug Deliv Rev. 2022;187: Article 114378. 35671882 10.1016/j.addr.2022.114378

[135] Strathdee SA, Hatfull GF, Mutalik VK, Schooley RT. Phage therapy: From biological mechanisms to future directions. Cell. 2023;186(1):17–31. 36608652 10.1016/j.cell.2022.11.017 PMC9827498

[136] Zuo T, Sun Y, Wan Y, Yeoh YK, Zhang F, Cheung CP, Chen N, Luo J, Wang W, Sung JJY, et al. Human-Gut-DNA Virome Variations across Geography, Ethnicity, and Urbanization. Cell Host Microbe. 2020;28(5):741–51.e4. 32910902 10.1016/j.chom.2020.08.005

[137] Mangold CA, Szpara ML. Persistent Infection with Herpes Simplex Virus 1 and Alzheimer’s Disease-A Call to Study How Variability in Both Virus and Host may Impact Disease. Viruses. 2019;11(10):966. 31635156 10.3390/v11100966 PMC6833100

[138] Bak IJ, Markham CH, Cook ML, Stevens JG. Intraaxonal transport of Herpes simplex virus in the rat central nervous system. Brain Res. 1977;136(3):415–429. 72587 10.1016/0006-8993(77)90067-1

[139] Jin BK, Belloni M, Conti B, Federoff HJ, Starr R, Son JH, Baker H, Joh TH. Prolonged in vivo gene expression driven by a tyrosine hydroxylase promoter in a defective herpes simplex virus amplicon vector. Hum Gene Ther. 1996;7(16):2015–2024. 8930662 10.1089/hum.1996.7.16-2015

[140] Song S, Wang Y, Bak SY, Lang P, Ullrey D, Neve RL, O’Malley KL, Geller AI. An HSV-1 vector containing the rat tyrosine hydroxylase promoter enhances both long-term and cell type-specific expression in the midbrain. J Neurochem. 1997;68(5):1792–1803. 9109503 10.1046/j.1471-4159.1997.68051792.x

[141] Wang Y, Yu L, Geller AI. Diverse stabilities of expression in the rat brain from different cellular promoters in a helper virus-free herpes simplex virus type 1 vector system. Hum Gene Ther. 1999;10(11):1763–1771. 10446916 10.1089/10430349950017446

[142] Rogers JH, Rhodes K, Roberts S, Raza M, Muir EM, Fawcett JW, Scarpini CG, Efstathiou S. A herpesvirus vector can transduce axotomized brain neurons. Exp Neurol. 2003;183(2):548–558. 14552896 10.1016/s0014-4886(03)00187-0

[143] Kim B, Kim YS, Li W, Kwon EB, Chung HS, Go Y, Choi JG. Ginsenoside Rg5, a potent agonist of Nrf2, inhibits HSV-1 infection-induced neuroinflammation by inhibiting oxidative stress and NF-kappaB activation. J Ginseng Res. 2024;48(4):384–394. 39036736 10.1016/j.jgr.2024.01.006 PMC11258381

[144] Chen SH, Damborsky JC, Wilson BC, Fannin RD, Ward JM, Gerrish KE, He B, Martin NP, Yakel JL. alpha7 nicotinic receptor activation mitigates herpes simplex virus type 1 infection in microglia cells. Antiviral Res. 2024;228: Article 105934. 38880195 10.1016/j.antiviral.2024.105934 PMC11250235

[145] Wang J, Qiao H, Wang Z, Zhao W, Chen T, Li B, Zhu L, Chen S, Gu L, Wu Y, et al. Rational Design and Acoustic Assembly of Human Cerebral Cortex-Like Microtissues from hiPSC-Derived Neural Progenitors and Neurons. Adv Mater. 2023;35(32): Article e2210631. 37170683 10.1002/adma.202210631

[146] Chen S, Zhou Y, Chen Y, Gu J. fastp: an ultra-fast all-in-one FASTQ preprocessor. Bioinformatics. 2018;34(17):i884–i890. 30423086 10.1093/bioinformatics/bty560 PMC6129281

[147] Dobin A, Davis CA, Schlesinger F, Drenkow J, Zaleski C, Jha S, Batut P, Chaisson M, Gingeras TR. STAR: ultrafast universal RNA-seq aligner. Bioinformatics. 2013;29(1):15–21. 23104886 10.1093/bioinformatics/bts635 PMC3530905

[148] Li H, Handsaker B, Wysoker A, Fennell T, Ruan J, Homer N, Marth G, Abecasis G, Durbin R, 1000 Genome Project Data Processing Subgroup. The Sequence Alignment/Map format and SAMtools. Bioinformatics. 2009;25(16):2078–2079. 19505943 10.1093/bioinformatics/btp352 PMC2723002

[149] Grabherr MG, Haas BJ, Yassour M, Levin JZ, Thompson DA, Amit I, Adiconis X, Fan L, Raychowdhury R, Zeng Q, et al. Full-length transcriptome assembly from RNA-Seq data without a reference genome. Nat Biotechnol. 2011;29(7):644–652. 21572440 10.1038/nbt.1883 PMC3571712

[150] Liao Y, Smyth GK, Shi W. The R package Rsubread is easier, faster, cheaper and better for alignment and quantification of RNA sequencing reads. Nucleic Acids Res. 2019;47(8): Article e47. 30783653 10.1093/nar/gkz114 PMC6486549

[151] Maere S, Heymans K, Kuiper M. BiNGO: a Cytoscape plugin to assess overrepresentation of gene ontology categories in biological networks. Bioinformatics. 2005;21(16):3448–3449. 15972284 10.1093/bioinformatics/bti551

[152] Shannon P, Markiel A, Ozier O, Baliga NS, Wang JT, Ramage D, Amin N, Schwikowski B, Ideker T. Cytoscape: a software environment for integrated models of biomolecular interaction networks. Genome Res. 2003;13(11):2498–2504. 14597658 10.1101/gr.1239303 PMC403769

[153] von Mering C, Huynen M, Jaeggi D, Schmidt S, Bork P, Snel B. STRING: a database of predicted functional associations between proteins. Nucleic Acids Res. 2003;31(1):258–261. 12519996 10.1093/nar/gkg034 PMC165481

[154] Karaba AH, Kopp SJ, Longnecker R. Herpesvirus entry mediator and nectin-1 mediate herpes simplex virus 1 infection of the murine cornea. J Virol. 2011;85(19):10041–10047. 21795335 10.1128/JVI.05445-11 PMC3196397

