# Supplementary material for: The Discovery of Phages in the Substantia Nigra and Its Implication for Parkinson’s Disease
Source: Research (Wash D C). 2025 Apr 30;8:0657. doi: 10.34133/research.0657 (PMC12041648; doi:10.34133/research.0657)
Supplement: Supplementary 1 — Figs. S1 to S14 Tables S1 to S10 [file research.0657.f1.zip › Supplemental Figures .docx]

**Legends of supplemental figures**

**Figure S1**


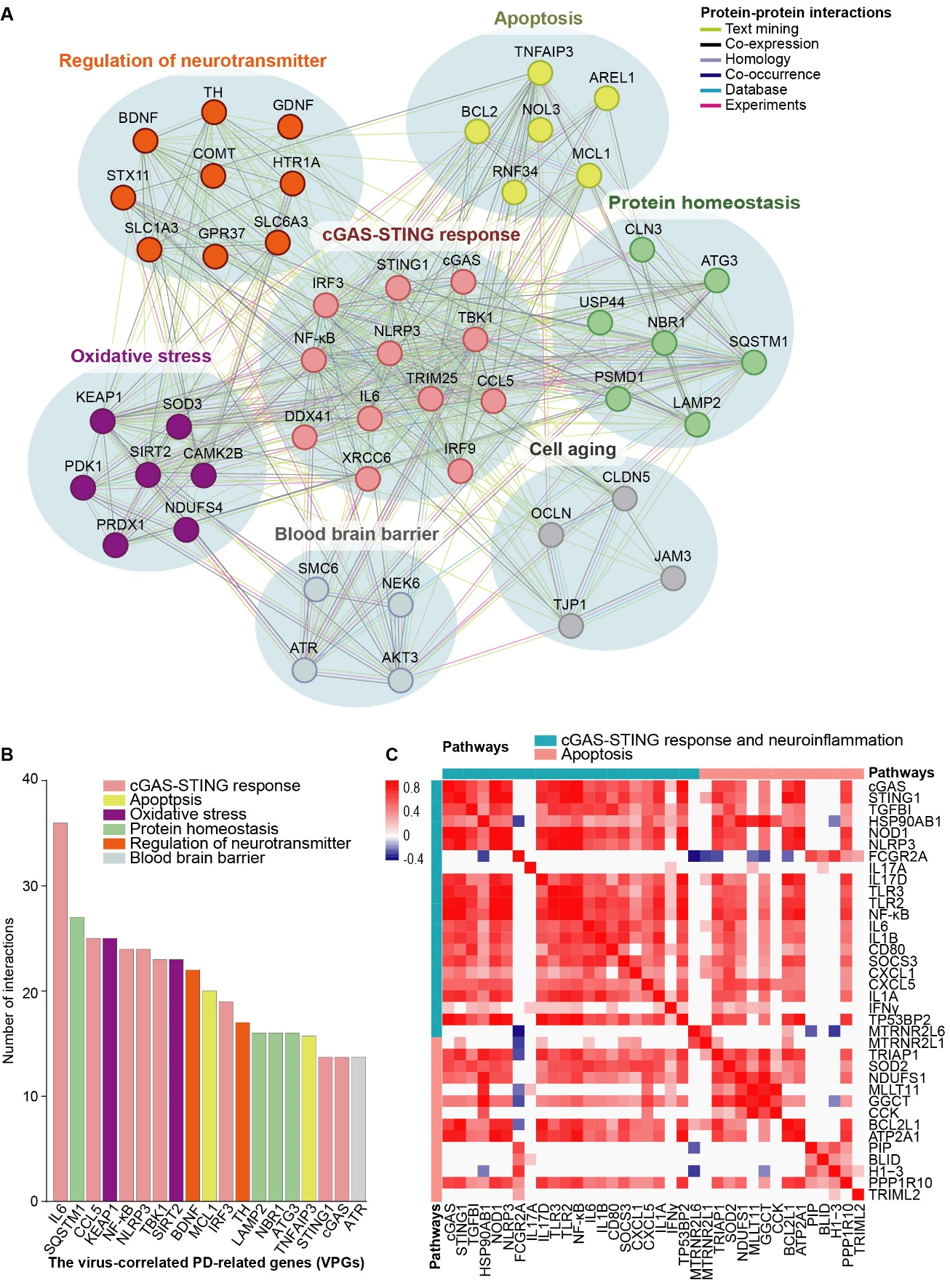


**Figure S1: PPI network analysis and correlation profiles between cGAS-STING response, neuroinflammation and apoptosis in the SN of PD patients**

(A) PPI network analysis of VPGs. The genes with more than two interactions are shown in the network. (B) Hub node genes determined by interaction numbers. A total of 15 genes with more than 10 interactions are shown in the bar plot. Abbreviation: Abbreviation: IL6, interleukin 6; SQSTM1, sequestosome 1; CCL5, C-C motif chemokine ligand 5; KEAP1, kelch like ECH associated protein 1; NF-κB, nuclear factor kappa B subunit 1; NLRP3, NLR family pyrin domain containing 3; TBK1, TANK binding kinase 1; SIRT2, sirtuin 2; BDNF, brain derived neurotrophic factor; MCL1, myeloid cell leukemia-1; IRF3, interferon regulatory factor 3; TH, tyrosine hydroxylase; LAMP2, lysosomal associated membrane protein 2; NBR1, neighbor of BRCA1 gene 1; ATG3, autophagy related 3; TNFAIP3, TNF alpha induced protein 3; STING1, stimulator of interferon response cGAMP interactor 1; cGAS, cyclic GMP-AMP synthase; ATR, ATR serine/threonine kinase. (C) Heatmap and clustering of VPGs based on their gene-gene pair correlations. Rows and columns represent human genes. (red: positive correlation; blue: negative correlation).

**Figure S2**


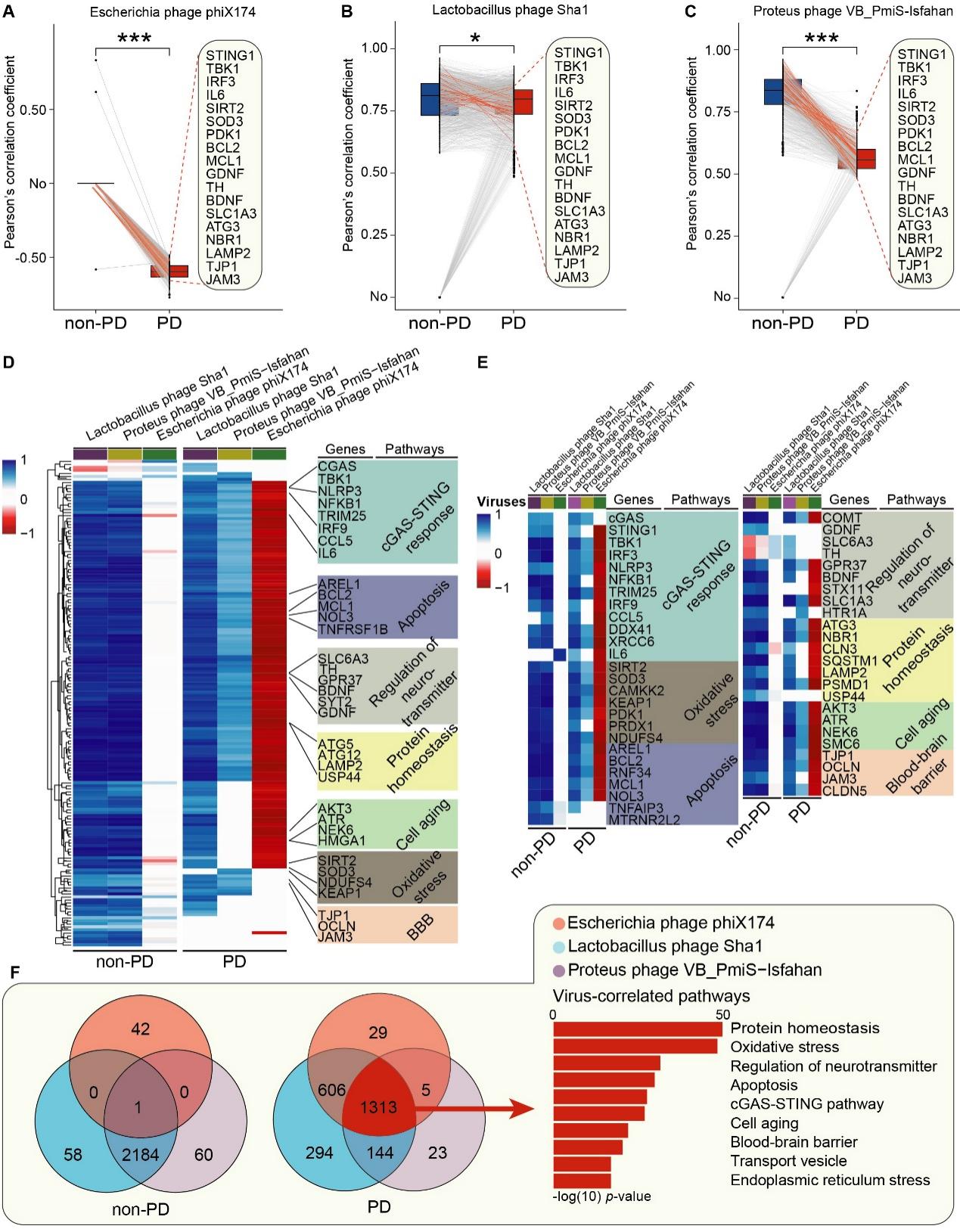


**Figure S2: A strong negative correlation between VRFC of the phage phix174 and PD-related human gene expression in the SN of PD patients**

(A, B, C) Paired boxplots showing changes of the correlations between viral RNA fragment counts (VRFC) of phages and PD-related gene expression in the SN of PD patients and non-PD individuals. Top and bottom edges represent the 1st and 3rd quartiles, respectively, the center line represents the median. The p values were calculated using the Wilcoxon matched-pairs test. *p value ＜ 0.05 and ***p value ＜ 0.001. (D) Hierarchical clustered heatmap of correlation profiles between VRFC of phages and PD-related human gene expression in the SN of PD patients and non-PD individuals. In the heat map, each column represents a phage, and each row represents a human gene. Red denotes negative correlation, and blue denotes positive correlation. (E) Heatmap of correlation profiles between VRFC of phages and expression of PD-related genes in the SN of PD patients and non-PD individuals. (F) The Venn diagram showing the overlapping PD-related genes and pathways among Escherichia phage phiX174, Lactobacillus phage Sha1 and Proteus phage VB_PmiS-Isfahan.

**Figure S3**


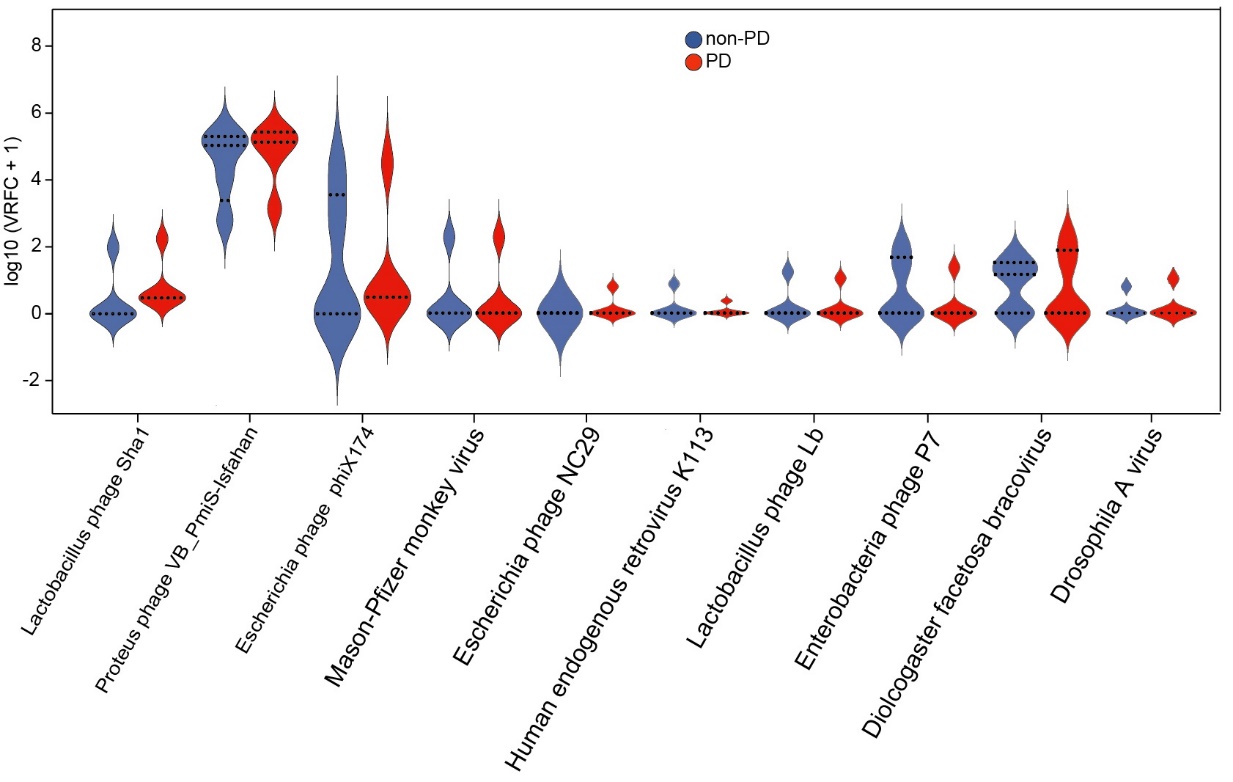


**Figure S3: Comparison of virobiota abundance in the SN of PD patients and non-PD individuals**

Violin plot showing the log_10_(VRFC + 1) of viruses in the SN of PD patients and non-PD individuals.

**Figure S4**


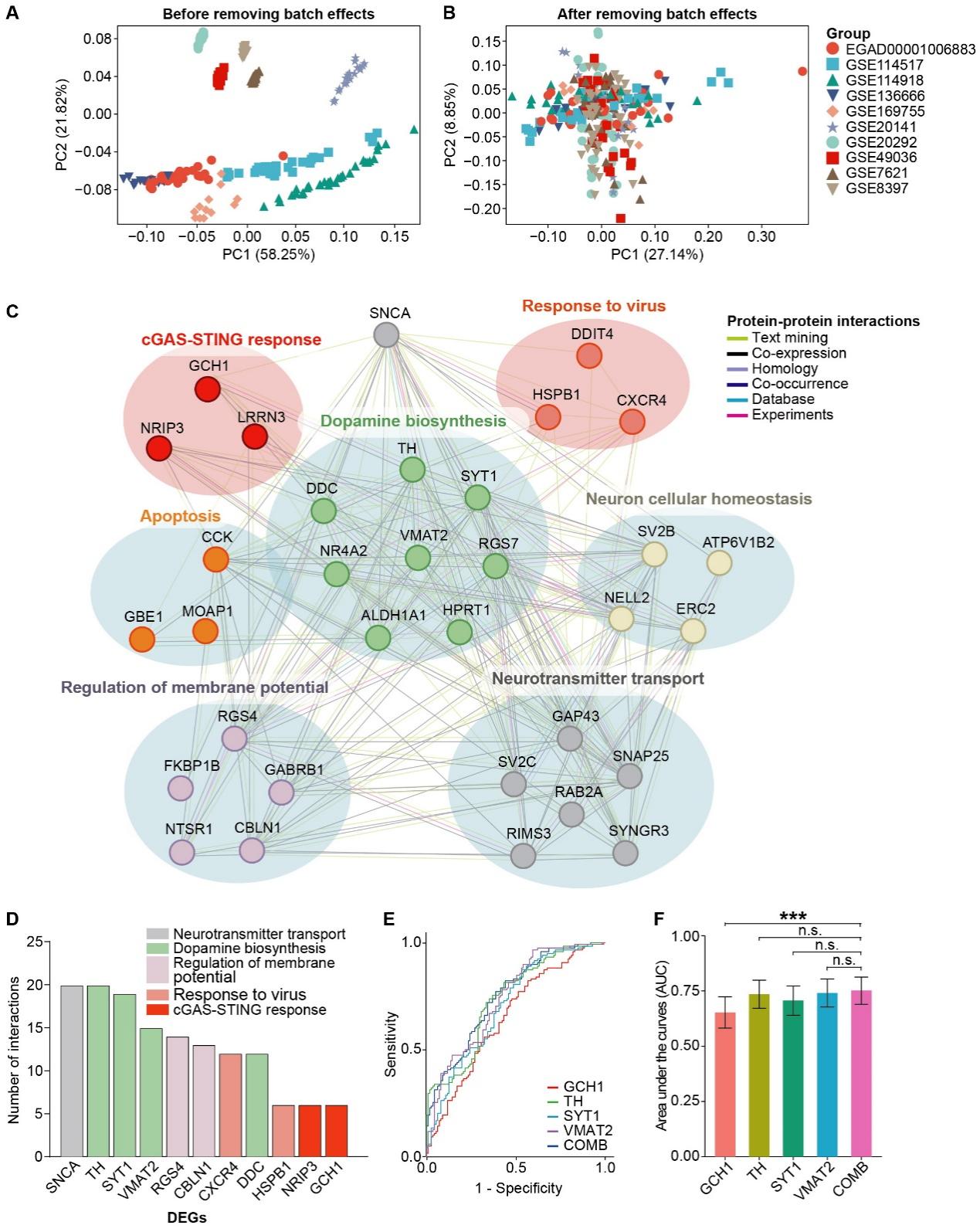


**Figure S4: PPI and ROC curve analysis of DEGs**

Principle component analysis (PCA) plot before removing batch effects (A) and after removing batch effects (B) for the RNA-seq datasets and microarray datasets. (C) PPI network analysis of DEGs. (D) Hub node genes determined by interaction numbers. A total of 24 genes with more than 5 interactions are shown in the bar plot. (E) ROC curve analysis of each and combination (COMB) of hub node genes. The curve of combination of four genes lies in the highest position than that of GCH1, TH, SYT1, and VMAT2 alone. (F) AUCs of each and combination of hub node genes. n.s. not significant; ****p* value ＜ 0.001.

**Figure S5**


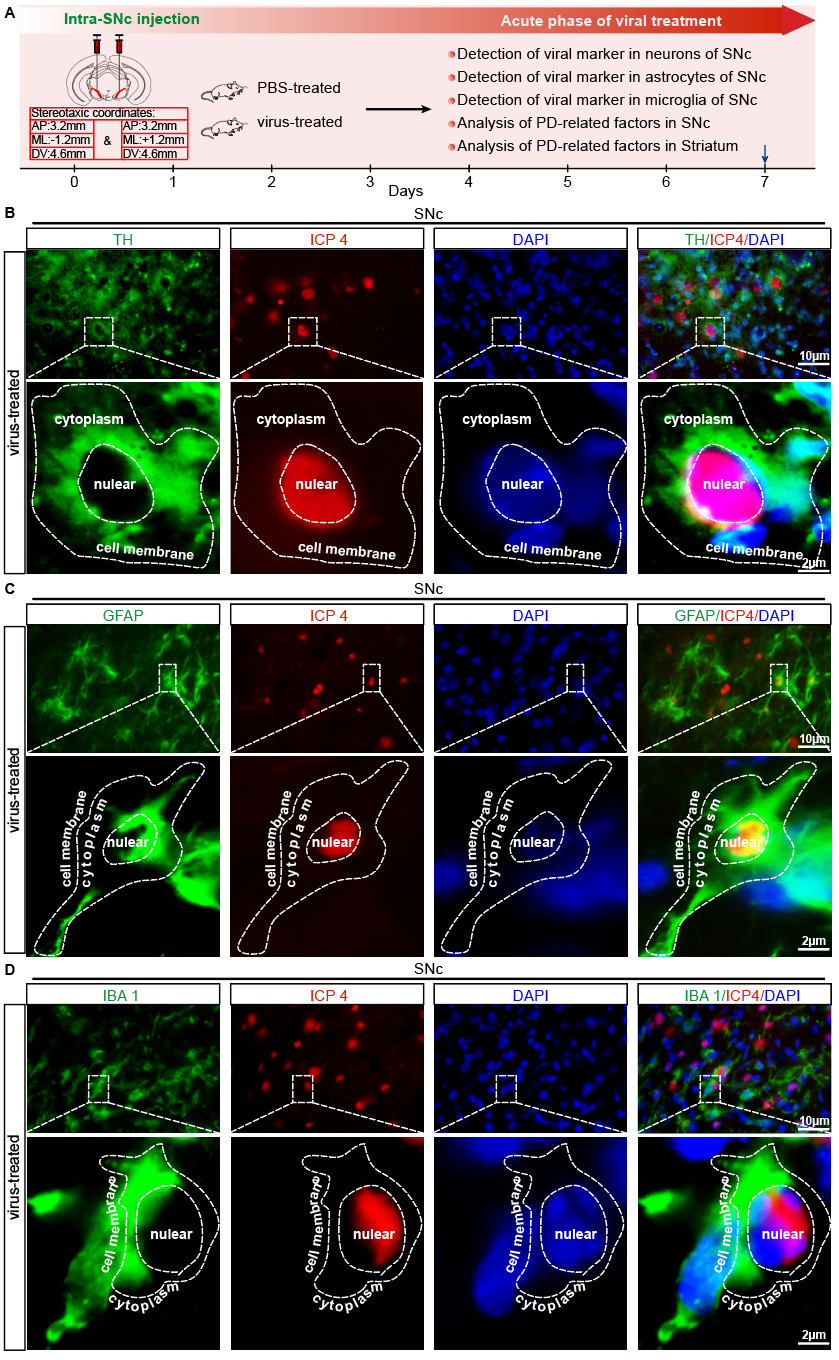


**Figure S5: Virus could affect SNc neural cells**

(A) The experimental workflow of parkinsonism detection during the acute phase of virus-treated. (B) Representative double immunostaining of TH with ICP 4 in SNc of virus-treated mice. Scale bar, 10 μm for low-magnification images and 2 μm for high-magnification images, respectively. (C) Representative double immunostaining of GFAP with ICP 4 in SNc of virus-treated mice. Scale bar, 10 μm for low-magnification images and 2 μm for high-magnification images, respectively. (D) Representative double immunostaining of IBA 1with ICP 4 in SNc of virus-treated mice. Scale bar, 10 μm for low-magnification images and 2 μm for high-magnification images, respectively.

**Figure S6**


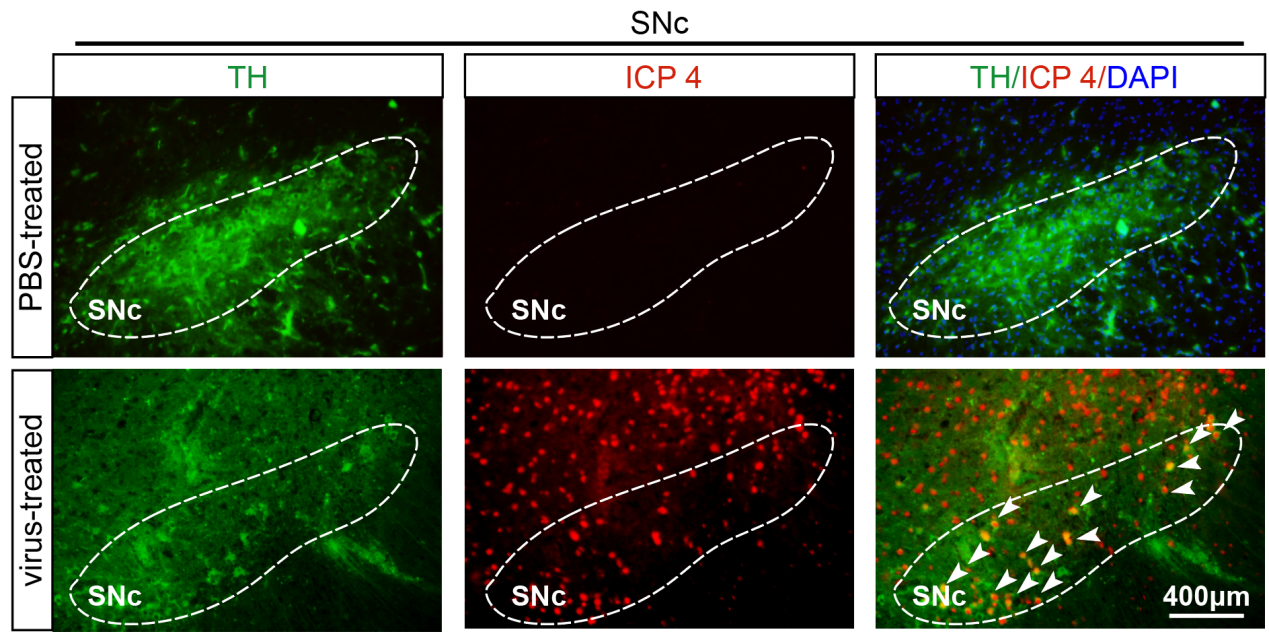


**Figure S6: Representative double immunostaining of TH with ICP 4 in SNc of PBS-treated and virus-treated mice. Scale bar, 400 μm. White arrow head indicates co-localization.**

**Figure S7**


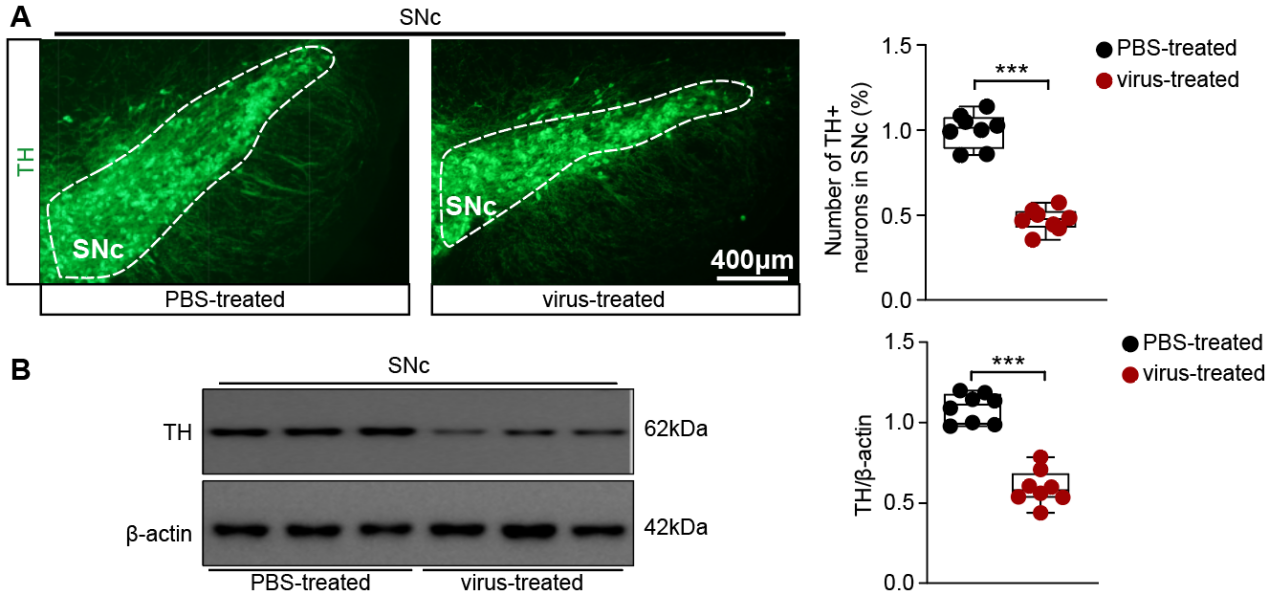


**Figure S7: A reduced TH immunoreactivity and a loss of dopaminergic neurons were observed in the SNc of virus-treated mice**

(A) Representative TH staining of SNc in PBS-treated and virus-treated mice, Scale bar, 400 μm. Unbiased stereological counts of TH^+^ cells in SNc of PBS-treated and virus-treated mice. Data are mean ± s.e.m.; n = 8 biologically independent animals; Student's t-test was used for statistical analysis, ****p* < 0.001. (B) Representative immunoblots of TH and β-actin in SNc of PBS-treated and virus-treated mice (cropped blot images are shown, see Figure S14 for full immunoblots). Quantification of TH protein levels in SNc. Data are mean ± s.e.m.; n = 8 biologically independent animals; Student's t-test was used for statistical analysis, ****p* < 0.001.

**Figure S8**


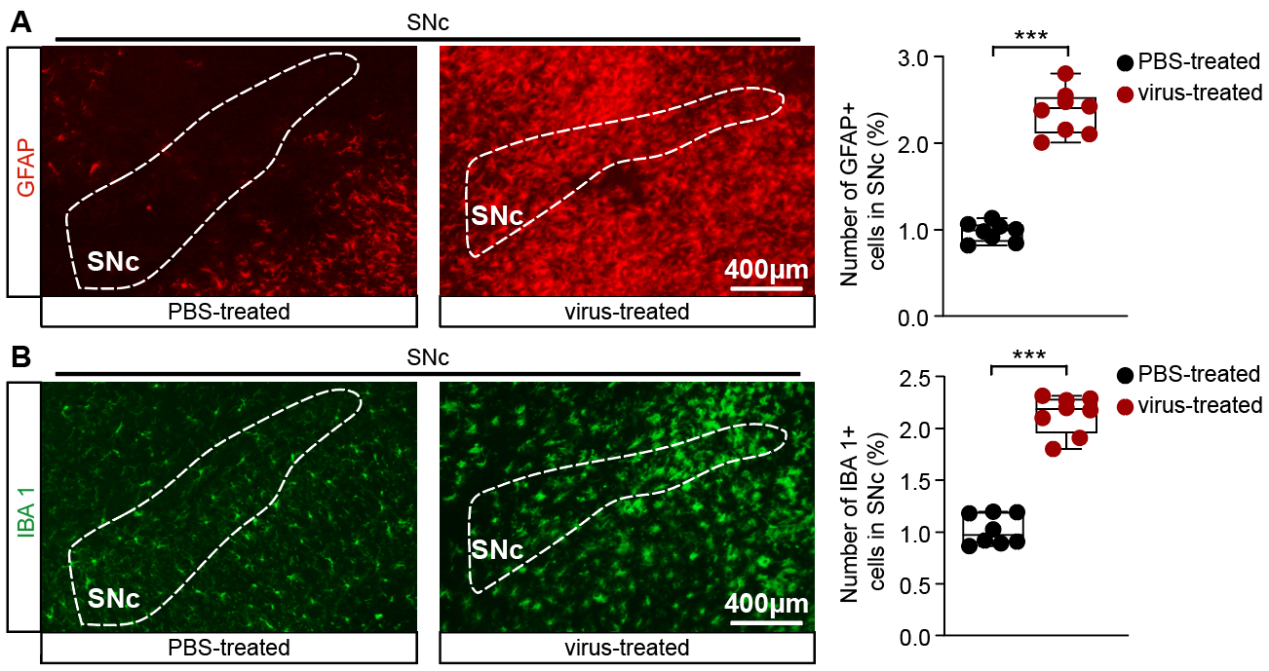


**Figure S8: An increased neuroinflammatory morphology of reactive astrocytes and microglia were observed were observed in the SNc of virus-treated mice.**

(A) Representative GFAP and IBA 1 staining of SNc in PBS-treated and virus-treated mice, Scale bar, 400 μm. Unbiased stereological counts of GFAP^+^ cells in SNc of PBS-treated and virus-treated mice. Data are mean ± s.e.m.; n = 8 biologically independent animals; Student's t-test was used for statistical analysis, ****p* < 0.001. (B) Representative IBA 1 staining of SNc in PBS-treated and virus-treated mice, Scale bar, 400 μm. Unbiased stereological counts of IBA 1^+^ cells in SNc of PBS-treated and virus-treated mice. Data are mean ± s.e.m.; n = 8 biologically independent animals; Student's t-test was used for statistical analysis, ****p* < 0.001.

**Figure S9**


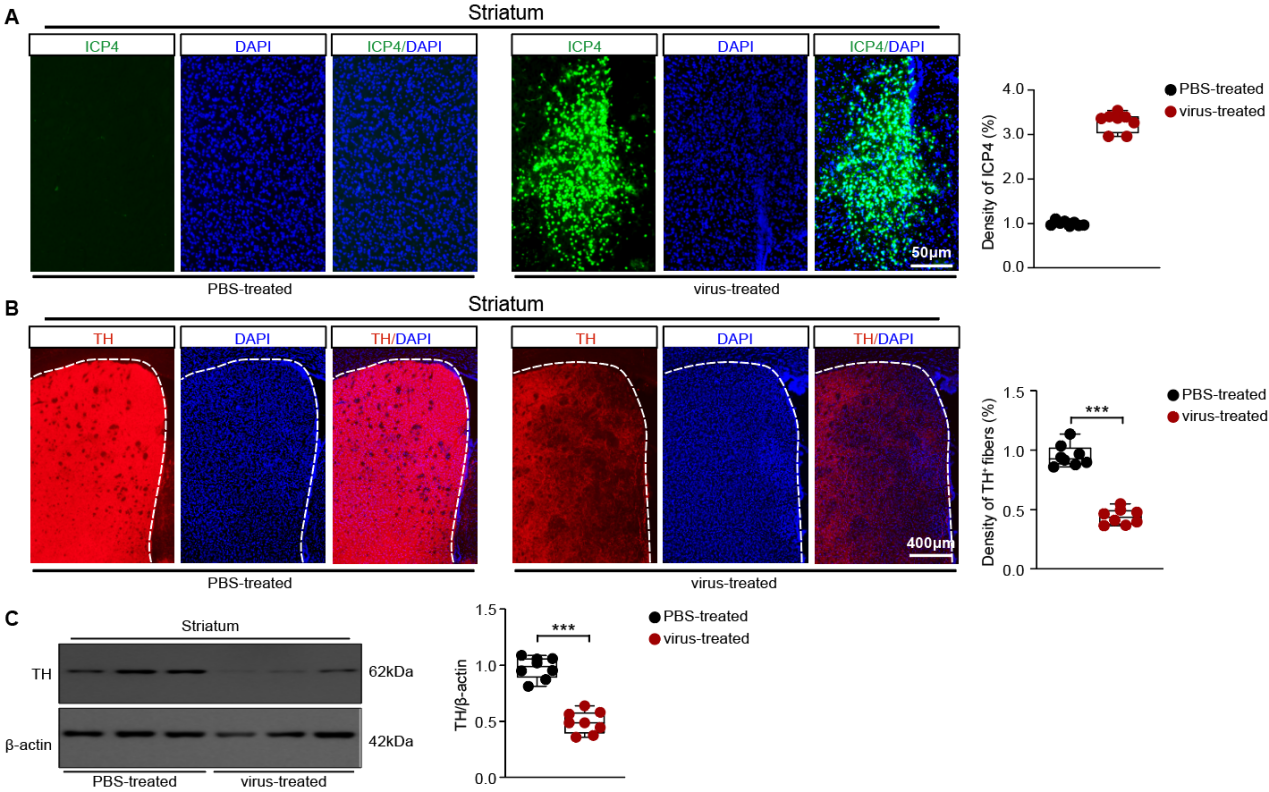


**Figure S9: Intra-SNc viral challenge pathologically affect neural cells in the striatum.**

(A) Representative images of ICP 4 in striatum of PBS-treated and virus-treated mice. Scale bar, 400 μm. Quantification of ICP 4 density. Data are mean ± s.e.m.; n = 8 biologically independent animals; Student's t-test was used for statistical analysis, ****p* < 0.001. (B) Representative images of TH staining in striatum, scale bar, 400 μm. Quantification of TH-positive striatal fiber density. Data are mean ± s.e.m.; n = 8 biologically independent animals; Student's t-test was used for statistical analysis, ****p* < 0.001. (C) Representative immunoblots of TH and β-actin in striatum of PBS-treated and virus-treated mice (cropped blot images are shown, see Figure S14 for full immunoblots). Quantification of TH protein levels in striatum. Data are mean ± s.e.m.; n = 8 biologically independent animals; Student's t-test was used for statistical analysis, ****p* < 0.001.

**Figure S10**


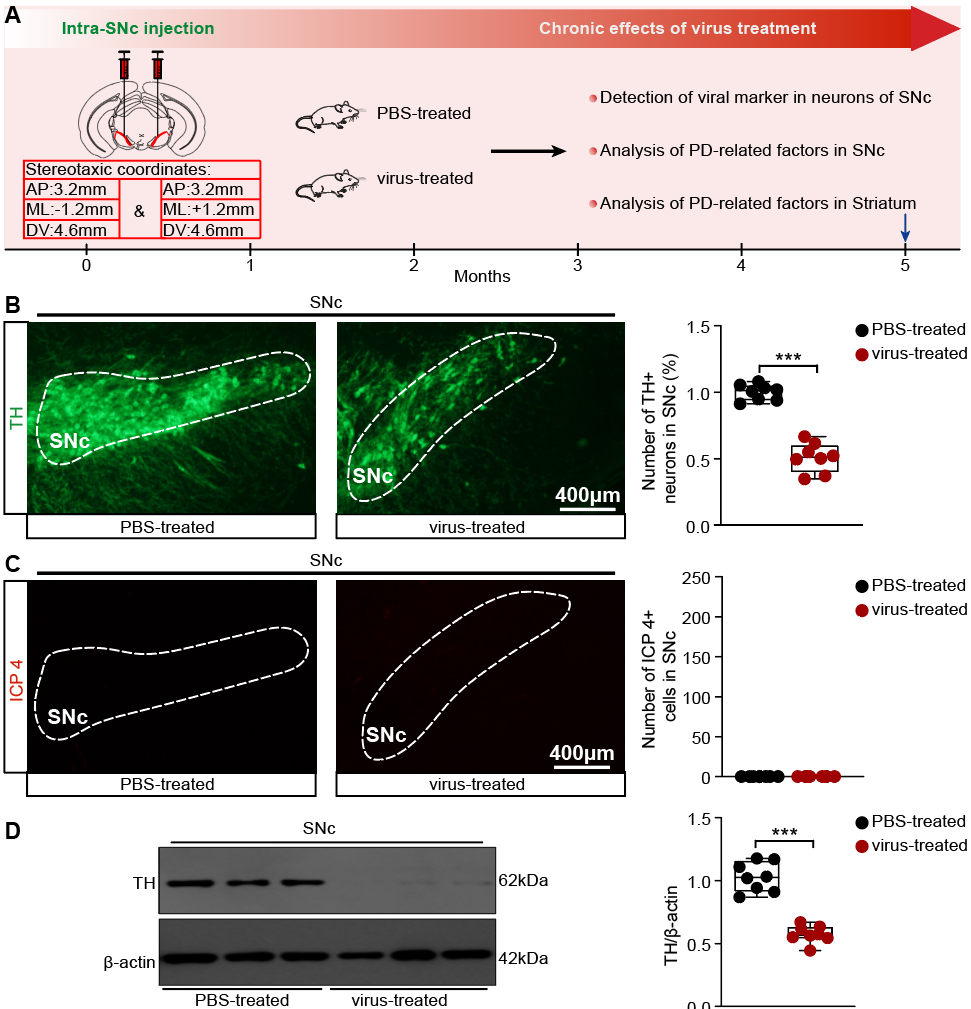


**Figure S10: The chronic viral treatment can induce the reduction of TH immunoreactivity and the loss of dopaminergic neurons in the SNc of mice.**

(A) The experimental workflow of parkinsonism detection during the chronic viral treatment. (B) Representative TH staining of SNc in PBS-treated and virus-treated mice, Scale bar, 400 μm. Unbiased stereological counts of TH^+^ cells in SNc of PBS-treated and virus-treated mice. Data are mean ± s.e.m.; n = 8 biologically independent animals; Student's t-test was used for statistical analysis, ****p* < 0.001.

(C) Representative immunostaining of ICP 4 in SNc of PBS-treated and virus-treated mice. Scale bar, 400 μm. Quantification of ICP 4 density. Data are mean ± s.e.m.; n = 8 biologically independent animals. (D) Representative immunoblots of TH and β-actin in SNc of PBS-treated and virus-treated mice (cropped blot images are shown, see Figure S14 for full immunoblots). Quantification of TH protein levels in SNc. Data are mean ± s.e.m.; n = 8 biologically independent animals; Student's t-test was used for statistical analysis, ****p* < 0.001.

**Figure S11**


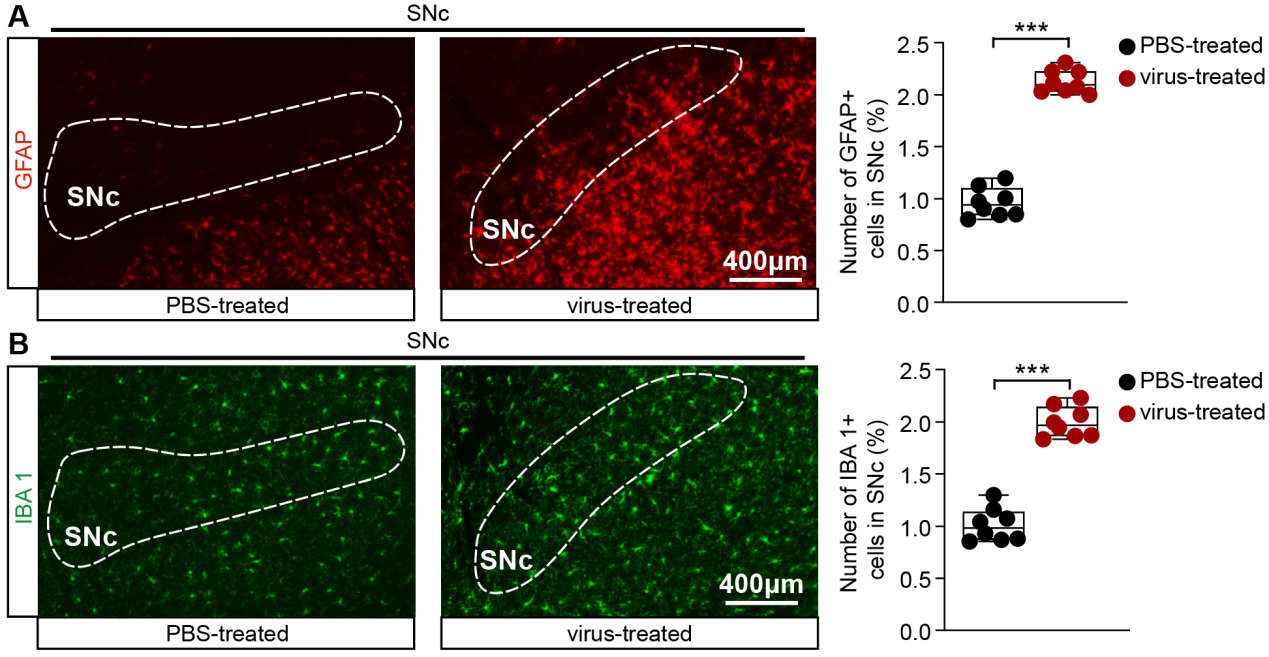


**Figure S11: The chronic viral treatment can induce the increase of neuroinflammatory morphology of reactive astrocytes and microglia in the SNc of mice.**

(A) Representative GFAP staining of SNc in PBS-treated and virus-treated mice, Scale bar, 400 μm. Unbiased stereological counts of GFAP^+^ cells in SNc of PBS-treated and virus-treated mice. Data are mean ± s.e.m.; n = 8 biologically independent animals; Student's t-test was used for statistical analysis, ****p* < 0.001. (B) Representative IBA 1 staining of SNc in PBS-treated and virus-treated mice, Scale bar, 400 μm. Unbiased stereological counts of IBA 1^+^ cells in SNc of PBS-treated and virus-treated mice. Data are mean ± s.e.m.; n = 8 biologically independent animals; Student's t-test was used for statistical analysis, ****p* < 0.001.

**Figure S12**


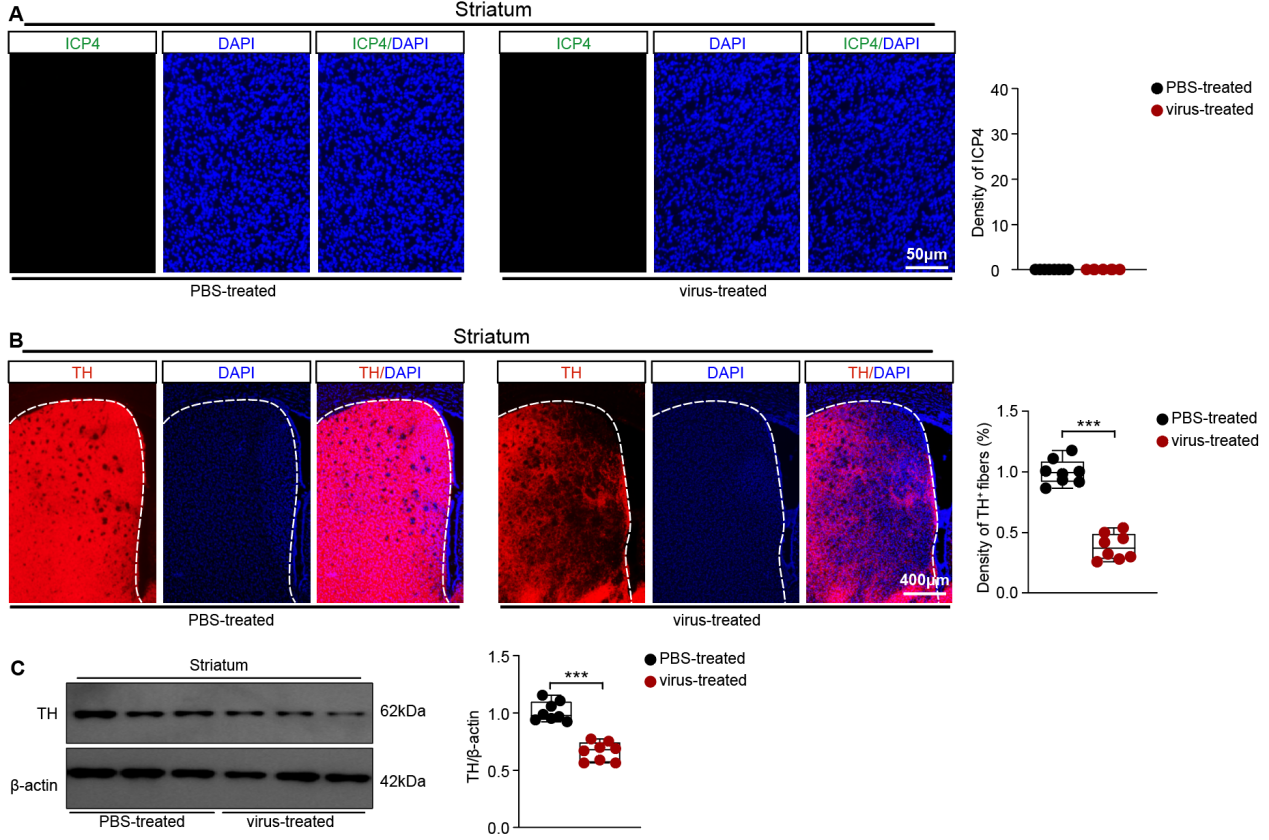


**Figure S12: The chronic viral treatment affects neural cells in the striatum.**

(A) Representative images of ICP 4 in striatum of PBS-treated and virus-treated mice. Scale bar, 400 μm. Quantification of ICP 4 density. Data are mean ± s.e.m.; n = 8 biologically independent animals. (B) Representative images of TH staining in striatum, scale bar, 400 μm. Quantification of TH-positive striatal fiber density. Data are mean ± s.e.m.; n = 8 biologically independent animals; Student's t-test was used for statistical analysis, ****p* < 0.001. (C) Representative immunoblots of TH and β-actin in striatum of PBS-treated and virus-treated mice (cropped blot images are shown, see Figure S14 for full immunoblots). Quantification of TH protein levels in striatum. Data are mean ± s.e.m.; n = 8 biologically independent animals; Student's t-test was used for statistical analysis, ****p* < 0.001.

**Figure S13**


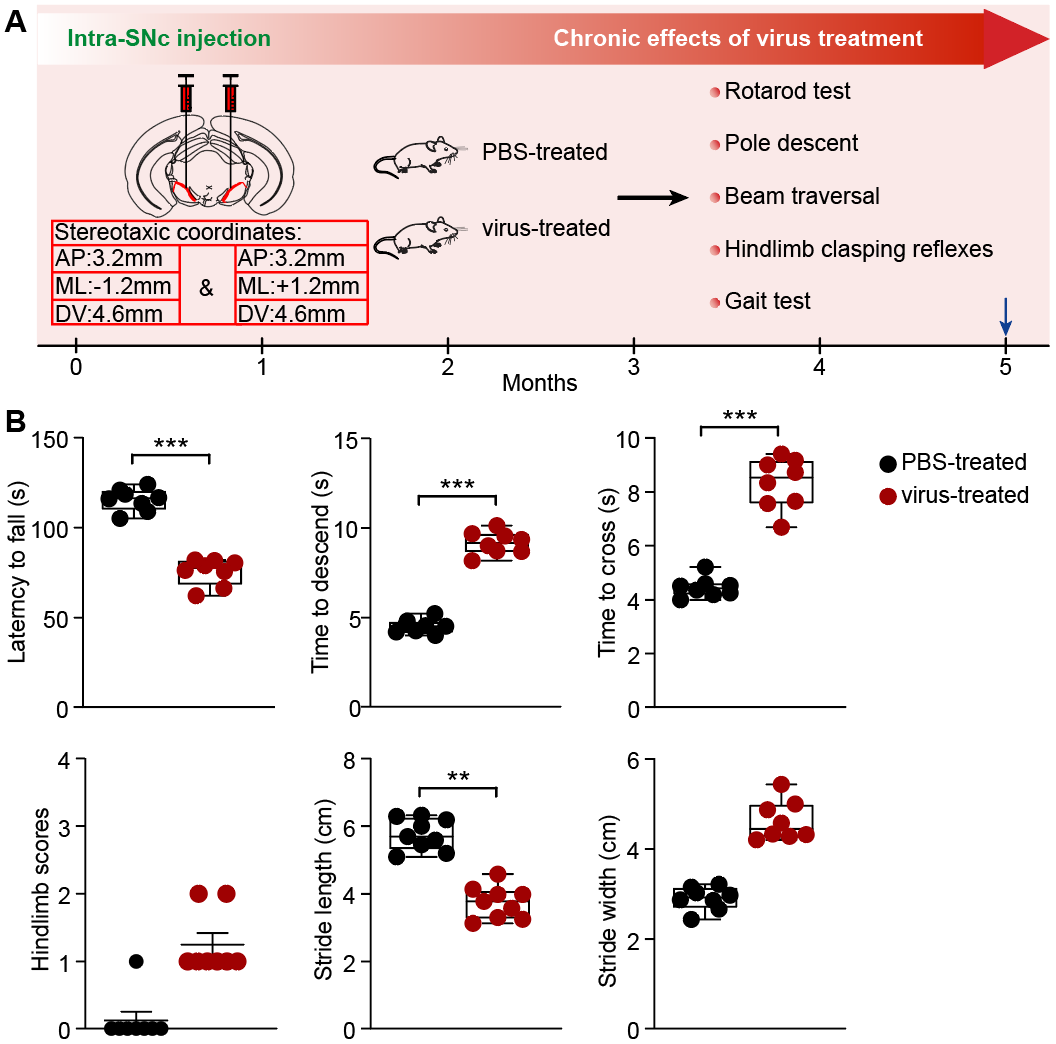


**Figure S13: The chronic viral treatment impaired motor coordination and balance.**

(A) The experimental workflow of motor coordination and balance detection during the chronic viral treatment. (B) Fall latency from an accelerating rotarod, time to traverse beam apparatus, time to descend pole, hindlimb clasping reflex score and gait analysis of PBS-treated and virus-treated mice. Data are mean ± s.e.m.; n = 8 biologically independent animals. The two-way ANOVA was used for statistical analysis followed by Tukey’s multiple comparisons test. ****p* < 0.001.

**Figure S14**


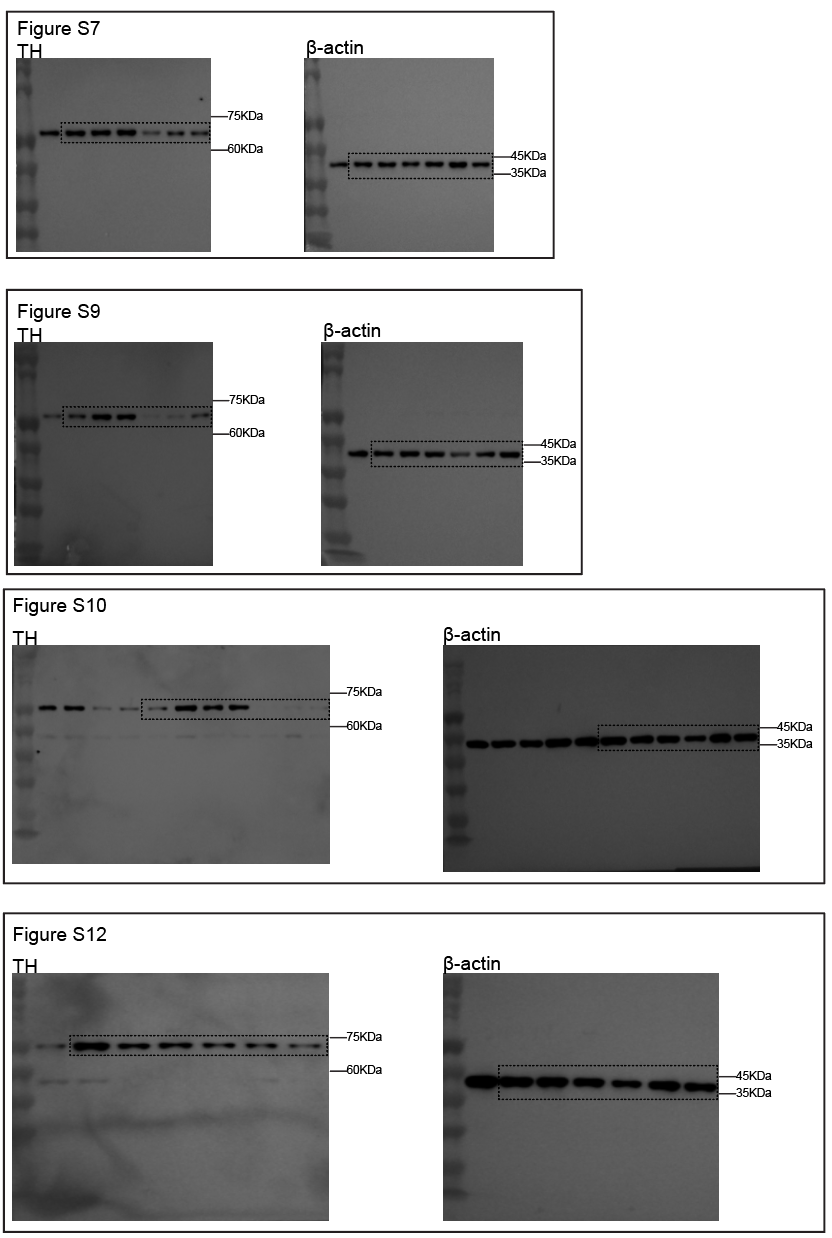


**Figure S14: Original full western blot images of Figure S7, S10 and S12.**
